# Long non-coding RNA GAS5 controls human embryonic stem cell self-renewal by maintaining NODAL signalling

**DOI:** 10.1038/ncomms13287

**Published:** 2016-11-04

**Authors:** Chen Xu, Yan Zhang, Qiaoling Wang, Zhenyu Xu, Junfeng Jiang, Yuping Gao, Minzhi Gao, Jiuhong Kang, Minjuan Wu, Jun Xiong, Kaihong Ji, Wen Yuan, Yue Wang, Houqi Liu

**Affiliations:** 1 Research Center of Developmental Biology, Second Military Medical University, 800th Xiangyin Road, Shanghai 200433, China; 2 Translational Medicine Center, Second Military Medical University, 800th Xiangyin Road, Shanghai 200433, China; 3 Department of Spine Surgery, Changzheng Hospital Affiliated to Second Military Medical University, 415th Feng Yang Road, Shanghai 200003, China; 4 Shanghai Key Laboratory for Assisted Reproduction and Reproductive Genetics, Center for Reprodutive Medcine, Renji Hospital, School of Medicine, Shanghai Jiao Tong University, Shanghai 200135, China; 5 School of Life Sciences and Technology, Tongji University, Shanghai 200092, China

## Abstract

Long non-coding RNAs (lncRNAs) are known players in the regulatory circuitry of the self-renewal in human embryonic stem cells (hESCs). However, most hESC-specific lncRNAs remain uncharacterized. Here we demonstrate that growth-arrest-specific transcript 5 (GAS5), a known tumour suppressor and growth arrest-related lncRNA, is highly expressed and directly regulated by pluripotency factors OCT4 and SOX2 in hESCs. Phenotypic analysis shows that GAS5 knockdown significantly impairs hESC self-renewal, but its overexpression significantly promotes hESC self-renewal. Using RNA sequencing and functional analysis, we demonstrate that GAS5 maintains NODAL signalling by protecting NODAL expression from miRNA-mediated degradation. Therefore, we propose that the above pluripotency factors, GAS5 and NODAL form a feed-forward signalling loop that maintains hESC self-renewal. As this regulatory function of GAS5 is stem cell specific, our findings also indicate that the functions of lncRNAs may vary in different cell types due to competing endogenous mechanisms.

## Abstract (web summary)

Long non-coding RNAs (lncRNA) are known to regulate human embryonic stem cell (hESC) self-renewal. Here, the authors identify lncRNA growth-arrest-specific transcript 5 (GAS5) that regulates pluripotency via modulation of NODAL signalling to maintain self-renewal in hESCs.

## Graphical abstract

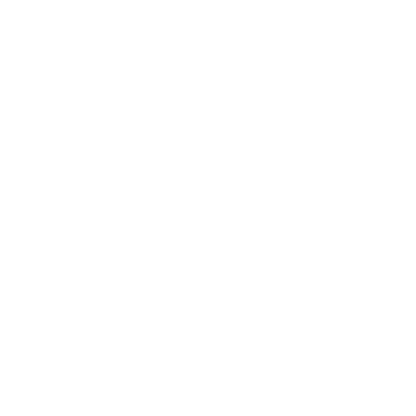

Embryonic stem cells (ESCs), which are derived from the inner cell mass, are pluripotent cells that possess unlimited proliferation potential and the ability to differentiate^1^. Pluripotency is tightly controlled by core transcription factors, signalling pathways and other regulators. Among several early attempts to reveal the signalling pathways that control ESC pluripotency, members of the transforming growth factor-β (TGFβ) superfamily were found to be crucial for the maintenance of the undifferentiated state^2^,^3^. Two signalling branches are involved in the self-renewal process in ESCs, the Nodal/Activin branch and the bone morphogenetic protein (BMP) branch. Downstream of these signalling pathways, NODAL/ACTIVIN signalling activates intracellular Smad2/3, whereas BMP signalling primarily activates Smad1/5/8 (ref. 4). However, compared with the BMP signalling, the regulation and function of NODAL/ACTIVIN signalling in hESCs is less been elucidated.

Non-coding RNAs (ncRNAs) were recently found to be important players in cell development, metabolism, differentiation and homoeostasis^5^. Of these, microRNAs (miRNAs) and long ncRNAs (>200 nucleotides, lncRNAs) are believed to play major regulatory roles in all multicellular organisms^6^,^7^. The roles of lncRNAs in hESCs are largely unclear; however, emerging evidence indicates that lncRNAs also play an essential role in regulating hESC-specific processes^8^,^9^,^10^. Many studies have shown that lncRNAs activate transcription, facilitate epigenetic modification and participate in post-transcriptional regulation in hESCs^11^. A recent work performed by our group showed that lncRNA-ROR functions as a sponge to protect the core transcription factors from miRNA binding^9^. However, the specific roles of lncRNAs in pluripotency regulation are still largely unknown.

In this study, taking advantage of high-throughput RNA sequencing technology, we investigate a set of highly expressed lncRNAs and identifiy that growth-arrest-specific transcript 5 (GAS5) correlates with hESC self-renewal. We show that GAS5 can increases OCT4, NANOG and SOX2 expression, and promotes the self-renewal of hESCs. We also show that GAS5 expression is directly controlled by the pluripotency factors OCT4 and SOX2, thus forming a circuit that promotes pluripotency. Through mechanism studies, we found that GAS5 attenuates miRNAs targeting the pluripotency-related TGFβ receptor family ligand NODAL, thus maintaining NODAL expression and promoting hESC self-renewal and pluripotency. Taken together, these findings demonstrate a new pluripotency regulatory circuit that functions via a miRNA competitive mechanism mediated by the lncRNA GAS5.

## Results

### GAS5 is highly expressed and controlled by OCT4/SOX2

**Figure 1:**
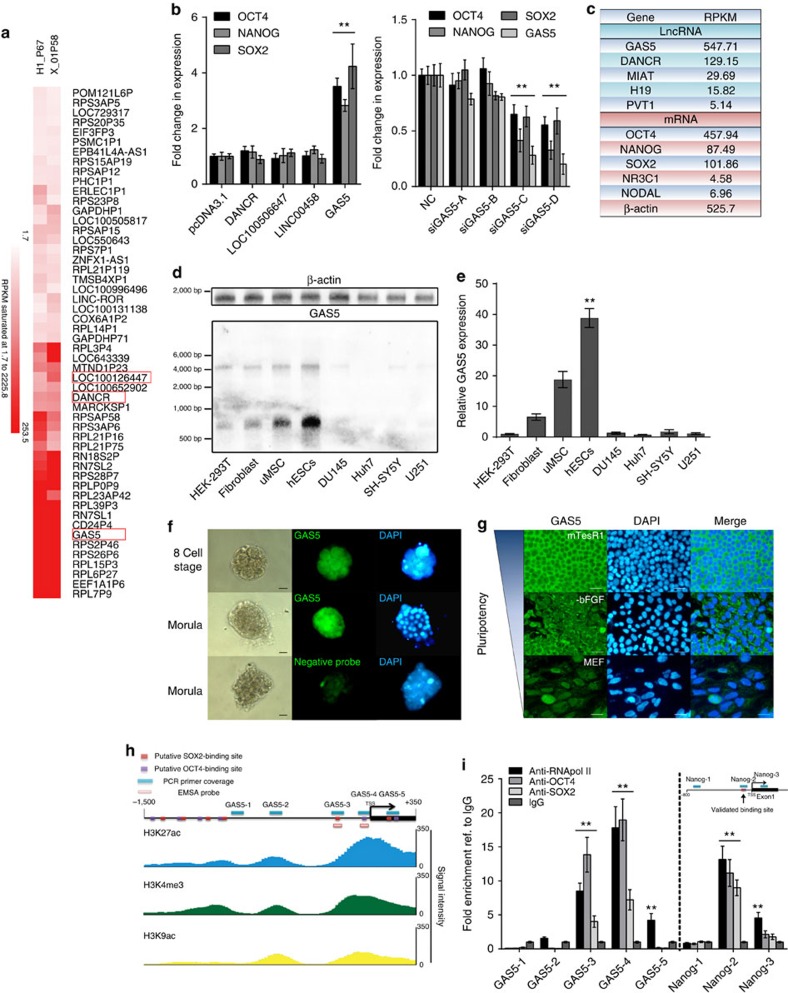
GAS5 is highly expressed in the cytoplasm and positively correlates with pluripotency. (**a**) Hierarchical clustering showing highly expressed lncRNAs in H1 or X-01 hESC cell lines. Candidate lncRNAs are highlighted in red square. (**b**) The left panel shows the mRNA level of OCT4, NANOG and SOX2 on lncRNA overexpression compared with an empty pcDNA3.1 vector control. Right panel shows the RNA level of pluripotency factors and GAS5 after transfected with different GAS5 short interfering RNAs (siRNAs). NC represents a scramble siRNA as negative control, GAS5-A/B/C/D represents different GAS5 knockdown siRNAs. **P<0.01, *t*-test, *n*=3. (**c**) RPKM value of several known lncRNAs and genes from the RNA sequencing were shown. (**d**) Northern blot analysis (*n*=2) using digoxingenin-labelled GAS5 anti-sense probe, the lower band indicates GAS5 transcript. Each lane is loaded with 2 μg total RNAs and a blot labelled with β-actin anti-sense probe served as loading control. (**e**) Relative RNA level of GAS5 in different cell types compared with HEK-293T. **P<0.01, *t*-test, *n*=3. (**f**) FISH analysis (*n*=2) of GAS5 in different human embryo stages using the digoxingenin-labelled GAS5 anti-sense probe. A sense strand probe was used as negative control probe. Scale bar, 50 μm. (**g**) FISH analysis of GAS5 in hESCs of different culture conditions (*n*=3). MTesR1 medium represents undifferentiated state, while −bFGF and MEF medium were used to induce hESCs differentiation. Scale bars, 100 μm. (**h**) A scheme of primers and probes used to detect the binding of pluripotency genes to the predicted promoter of *GAS5*. Lower part shows the coordinate active histone modification state from ENCODE ChIP-sequencing data. (**i**) ChIP analysis of the binding of OCT4 and SOX2 to *GAS5* promoter using the indicated antibody with the primers in **h**. The analysis of NANOG promoter was shown as positive control. Data were first normalized to input then compared with IgG groups. **P<0.01, *t*-test, *n*=3. Error bars represent s.d. of the indicated experiment replicates. RNA level of β-actin served as internal reference for qPCR. See also Supplementary Fig. 1.

To identify lncRNAs that affect the pluripotency of hESCs, we first searched for highly expressed candidate lncRNAs via high-throughput RNA sequencing in two hESC lines (H1 and X-01). Of the annotated lncRNAs that we identified, the highly expressed GAS5 drew our interest (Fig. 1a). This formerly identified tumour suppressor and growth arrest lncRNA is extremely enriched in hESCs. We overexpressed some of the highly expressed lncRNAs and found only GAS5 remarkably and dose-dependently increased pluripotency-related OCT4, NANOG and SOX2 expression (*t*-test, *P*<0.01 Fig. 1b left panel; Supplementary Fig. 1a), while GAS5 silencing significant reduced the expression of these pluripotency genes in hESCs (*t*-test, *P*<0.01 Fig. 1b right panel; Supplementary Fig. 1b).

Of the lncRNAs reported in hESCs^12^,^13^,^14^,^15^, GAS5 is the most highly expressed, with an expression level similar to that of OCT4 (Fig. 1c). We performed northern blotting and quantitative PCR (qPCR) analyses to determine GAS5's expression in other cell types; however, results showed the expression in hESCs remains the highest (Fig. 1d,e). To demonstrate the dynamic changes in GAS5 expression during both embryonic development and hESC differentiation, we used fluorescence *in situ* hybridization (FISH). We found that GAS5 levels increased along with human embryo cleavage and that GAS5 was abundantly expressed in the cytoplasm of hESCs (Fig. 1f,g). However, its expression decreased rapidly under various differentiation conditions (Fig. 1g and Supplementary Fig. 1d), which is similar to the previous reports demonstrating that GAS5 expression changes during mouse embryonic development^16^,^17^ and localization of GAS5 in HeLa cells^18^. We thus hypothesized that pluripotency factors are related to the GAS5 expression. To test this hypothesis, we overexpressed OCT4, NANOG and SOX2 individually, and found that GAS5 was significantly upregulated only when OCT4 and SOX2 were overexpressed (Supplementary Fig. 1e). We performed a chromatin immunoprecipitation (ChIP) assay along with an electrophoretic mobility shift assay (EMSA) assay to confirm the binding of OCT4 and SOX2 to the GAS5 promoter at sites specified by the JASPAR database and corresponding histone modifications as specified by ENCODE (Fig. 1h; Supplementary Fig. 1f; Supplementary Data 1). The results confirmed the binding of OCT4 and SOX2 to the region extending from −400 bp to the TSS (Transcription start site), where the histone markers of transcriptional activation were most highly expressed (Fig. 1I; Supplementary Fig. 1g–i). These findings indicate that *GAS5* transcription is directly controlled by OCT4 and SOX2, and may explain why *GAS5* is more highly expressed in hESCs than in other cell types.

### GAS5 is essential for hESC self-renewal

**Figure 2:**
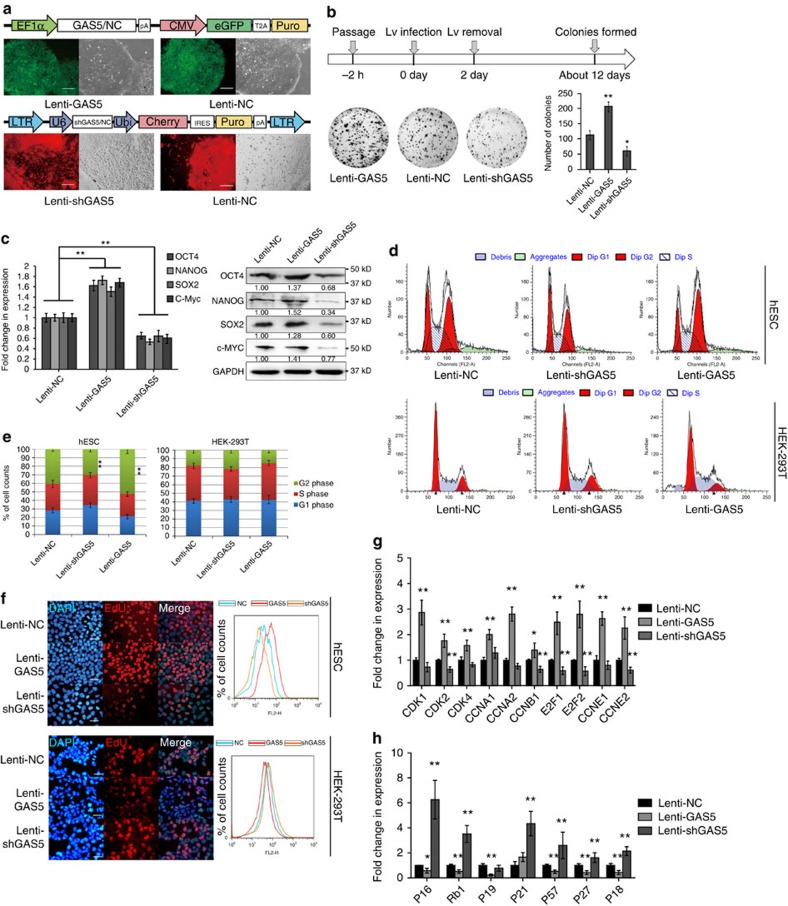
GAS5 is essential for hESC self-renewal. (**a**) A scheme of lentiviral vectors constructed. The lower panel shows the transefected cell colonies obtained after puromycin selection in fluorescence microscopy (*n*=3). Lenti-NC represents control lentivirus, Lenti-GAS5 represents GAS5-overexpressing lentivirus and Lenti-shGAS5 represents short hairpin knockdown lentivirus. Scale bar, 500 μm. (**b**) Colony formation assay of GAS5 overexpression or knockdown hESCs. The scheme of assay is shown above, and the quantitation of colonies is shown in the lower right panel. **P<0.01, *t*-test, *n*=3. (**c**) qPCR and western blot analysis of puipotency genes in different lentivirus transfected cells. **P<0.01, one-way analysis of variance, *n*=3. (**d**,**e**) The cell cycle analysis (*n*=3) of different stably expressed hESCs and HEK-293T cells (**d**), the quantification of cell cycle phases in different stably expressed hESCs and HEK-293T cells are shown in **e**. Ratio of G2 phase cells were compared for significance as indicated. **P<0.01, *t*-test, *n*=3. (**f**) Representative images of EdU analysis (*n*=3) and flow cytometry quantification (*n*=3) of different expressed hESCs and HEK-293T cells. (**g**,**h**) The mRNA level of positive regulators (**g**) and negative regulators (**h**) of cell cycle in different stably expressed hESCs. *P<0.05, **P<0.01, *t*-test, *n*=3. Error bars represent s.d. of the indicated experiment replicates. RNA level of β-actin served as internal reference for qPCR. See also Supplementary Fig. 2.

To determine the role of GAS5 in hESC self-renewal, we constructed lentiviral vectors expressing either GAS5 or GAS5 short hairpin RNA (Fig. 2a; Supplementary Fig. 2a). Knockdown of GAS5 (Lenti-shGAS5) in hESCs resulted in more spontaneous differentiation (Fig. 2a, lower panels), while overexpression of GAS5 resulted in the formation of denser and larger colonies (Lenti-GAS5, Supplementary Fig. 2b). This observation was confirmed via colony formation assay (Fig. 2b), where more and larger colonies formed in Lenti-GAS5 hESCs than in those that received an empty vector (negative control, NC) group, and fewer colonies formed in Lenti-shGAS5 hESCs. By examining pluripotency gene expression, we found that Lenti-shGAS5 cells exhibited decreased pluripotency gene expression, while Lenti-GAS5 cells exhibited increased pluripotency gene expression (Fig. 2c). This effect was partially reversed by knockdown of GAS5 in Lenti-GAS5 hESCs or overexpression of GAS5 in Lenti-shGAS5 cells (Supplementary Fig. 2c).

We next examined the impact of GAS5 on the hESC cell cycle. The proportion of G2/M phase cells was decreased in Lenti-shGAS5 cells, and the proportion of G1/G0 phase cells was significantly increased (*t*-test, *P*<0.01). Contrasting findings were noted in Lenti-GAS5 cells, indicating that GAS5 expression influences the hESC cell cycle (Fig. 2d,e; Supplementary Fig. 2d). We also employed an EdU (5-ethynyl-2′-deoxyuridine) incorporation assay to quantitatively assess cell proliferation. As expected, Lenti-GAS5 cells showed greater EdU incorporation than NC cells, whereas Lenti-shGAS5 cells showed less incorporation than NC cells (Fig. 2f). In addition, we noted that Cyclin and CDK gene expression was upregulated and that negative cell cycle regulator expression was downregulated in Lenti-GAS5 cells. Contrasting findings were observed in Lenti-shGAS5 cells (Fig. 2g,h). Moreover, we performed a cell cycle synchronization assay to exclude the influence of hESC heterogeneity (Supplementary Fig. 2d). The results indicated that Lenti-shGAS5 cells recovered more slowly, while Lenti-GAS5 hESCs recovered more quickly. Together, these results confirmed the essential role of GAS5 in sustaining hESC self-renewal.

To determine the function of GAS5 in differentiation, we employed an fetal bovine serum (FBS)-induced differentiation model. We found that Lenti-GAS5 hESCs expressed fewer differentiation markers than control cells and Lenti-shGAS5 cells (Supplementary Fig. 2f). Alkaline phosphatase (AP) staining revealed better preservation of pluripotency in Lenti-GAS5 cells than in other cells (Supplementary Fig. 2g). Embryo body formation assays also showed that Lenti-GAS5 cells formed denser embryoid body (EB) spheres, while Lenti-shGAS5 cells formed smaller and fewer EB spheres (Supplementary Fig. 2b, lower panels). These results are indicative of the essential role of GAS5 in maintaining hESC pluripotency and self-renewal.

However, GAS5 exerted growth-arrest effects in HEK-293T cells (Fig. 2d–f), which have been observed in other cell lines^19^,^20^,^21^,^22^, indicating that the abovementioned effects of GAS5 may be hESC specific.

### NODAL–SMAD signalling is activated by GAS5 in hESCs

**Figure 3:**
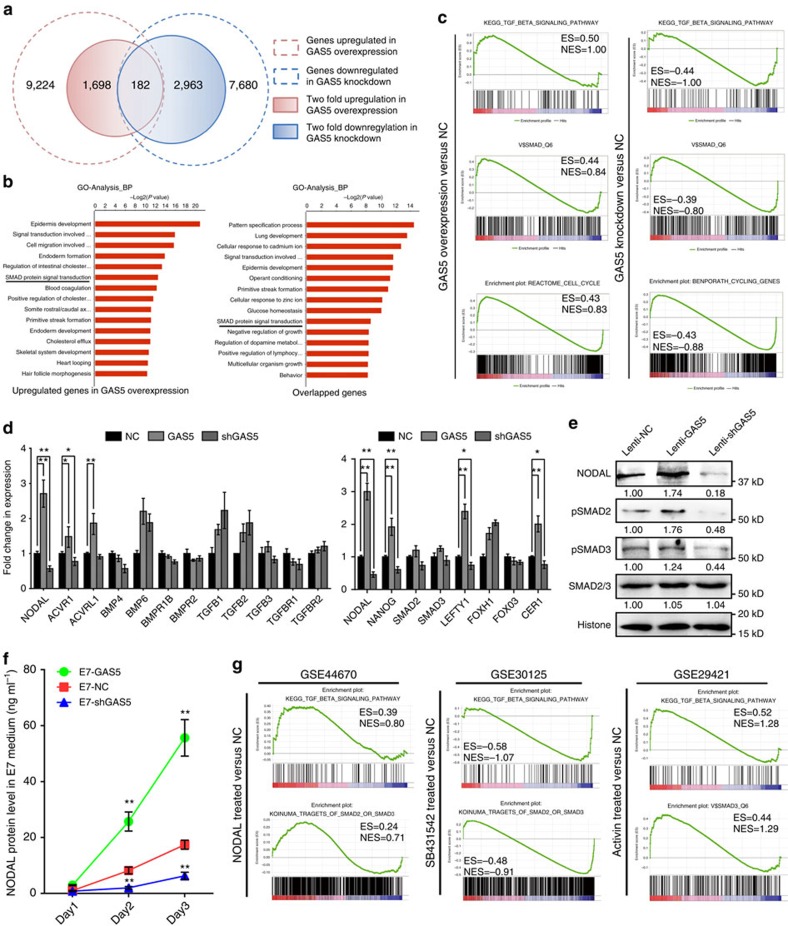
Global mRNA analysis revealed NODAL–SMAD signalling is controlled by GAS5. (**a**) High-throughput RNA sequencing of GAS5 stably overexpressed or knockdown hESCs. The graph depicts the number of GAS5 co-expressed genes generated from the sequencing. (**b**) Gene ontology analysis of the differentially expressed genes. The left panel used 1,698 genes with twofold up regulation in expression and the right panel used 182 genes in the intersection part in **a**. (**c**) Gene set enrichment analysis of gene signatures (GSEA) in GAS5 overexpressed hESCs versus control group, and GAS5 knockdown hESCs versus control group. ES represents enrichment score and NES represents normalized enrichment score in each category. Higher score represents higher possibility that the treatment has positive effect on such gene category. (**d**) qPCR analysis of key genes and receptors in the TGFβ families in GAS5 overexpressed or knockdown hESCs (left panel). The right panel shows qPCR analysis of genes involved in the NODAL signalling in GAS5 overexpressed or knockdown hESCs. *P<0.05, **P<0.01, *t*-test, *n*=3. (**e**) Western blot (*n*=3) showing changes of NODAL, SMAD2/3 and p-SMAD2/3 in GAS5 overexpressed or knockdown hESCs. (**f**) ELISA analysis of NODAL in the indicated cell culture supernatant. E7 is a chemical defined and serum-free culture medium used for analysing the net NODAL production of hESCs that influenced by GAS5 overexpression or knockdown. The ingredient of E7 medium does not contain TGFβ, NODAL nor Activin. **P<0.01, *t*-test, *n*=3. (**g**) GSEA gene signatures of microarray or high-throughput sequencing data that involve the activation or inhibition of NODAL or Activin signaling. SB431542, an ALK4/7 inhibitor. Error bars represent s.d. of the indicated experiment replicates. RNA level of β-actin served as internal reference for qPCR. See also Supplementary Fig. 3.

To determine the mechanism underlying the function of GAS5 in hESC self-renewal, we analysed global gene expression via RNA sequencing. We identified 1698 upregulated genes (fold change ≥2) and 2,963 downregulated genes (fold change ≥2) in the GAS5 knockdown group on GAS5 overexpression (Fig. 3a; Supplementary Fig. 3a). Here we first analysed the GAS5 co-expressed genes (overlapped region in Fig.3a) using gene ontology analysis, we found that many of the differentially expressed genes are related to SMAD signalling (Fig. 3b; Supplementary Data 2) and that many of the genes downregulated in Lenti-shGAS5 hESCs are related primarily to the cell cycle (Supplementary Fig. 3b). Gene set enrichment analysis (GSEA) and pathway analyses also demonstrated enrichment of TGFβ signalling in Lenti-GAS5 hESCs (Fig. 3c; Supplementary Fig. 3c). In contrast, the changes in global gene expression in Lenti-shGAS5 hESCs were negatively enriched with respect to TGFβ signalling and SMAD-binding sites (Fig. 3c). However, the analysis results of GAS5 inversely correlated genes (downregulated in GAS5 overexpression and upregulated in GAS5 knockdown, Supplementary Fig. 3d,e) showed none pluripotency-related terms are enriched. Thus indicates GAS5 may played a role in TGFβ–SMAD signalling in hESCs.

TGFβ signalling involves various ligands and receptors^23^,^24^. To identify specific genes affected by GAS5, we screened various TGFβ signalling-related genes and found a significant correlation between NODAL and GAS5 expression (Fig. 3d; Supplementary Fig. 3f). We noted elevated NODAL protein expression and increased SMAD2/3 phosphorylation on GAS5 overexpression, as well as contrasting results on GAS5 knockdown, and no significant changes in the expression of other key signalling genes (Fig. 3e; Supplementary Fig. 3g). Because NODAL can be secreted, we measured net NODAL production in serum free, TGFβ/NODAL/Activin-free medium E7. As expected, Lenti-GAS5 cells produced more NODAL in the supernatant than control cells, whereas GAS5 knockdown cells produced less than half of the normal amount (Fig. 3f).

To find additional evidence indicating that the function of GAS5 is related to NODAL signalling, we chose three hESC-related or embryonic carcinoma-related microarray data sets from the GEO database^25^,^26^,^27^ that were treated with rhNODAL, a NODAL inhibitor or Activin (a cytokine that shares receptors with NODAL) and analysed gene enrichment in the TGFβ and SMAD-binding subsets via GSEA. The rhNODAL- and Activin-treated samples exhibited enrichment in the TGFβ and SMAD-binding-related subsets similar to that exhibited in Lenti-GAS5 cells, while NODAL inhibition (SB431542, an inhibitor of NODAL receptors) produced results more closely resembling those produced in Lenti-shGAS5 hESCs (Fig. 3g; Supplementary Fig. 3h). Together, these data provide evidence that GAS5 activates NODAL signalling by promoting NODAL secretion and that activation of NODAL signalling results in phenotypes similar to that of GAS5.

### NODAL is required for GAS5 to promote hESC self-renewal

**Figure 4:**
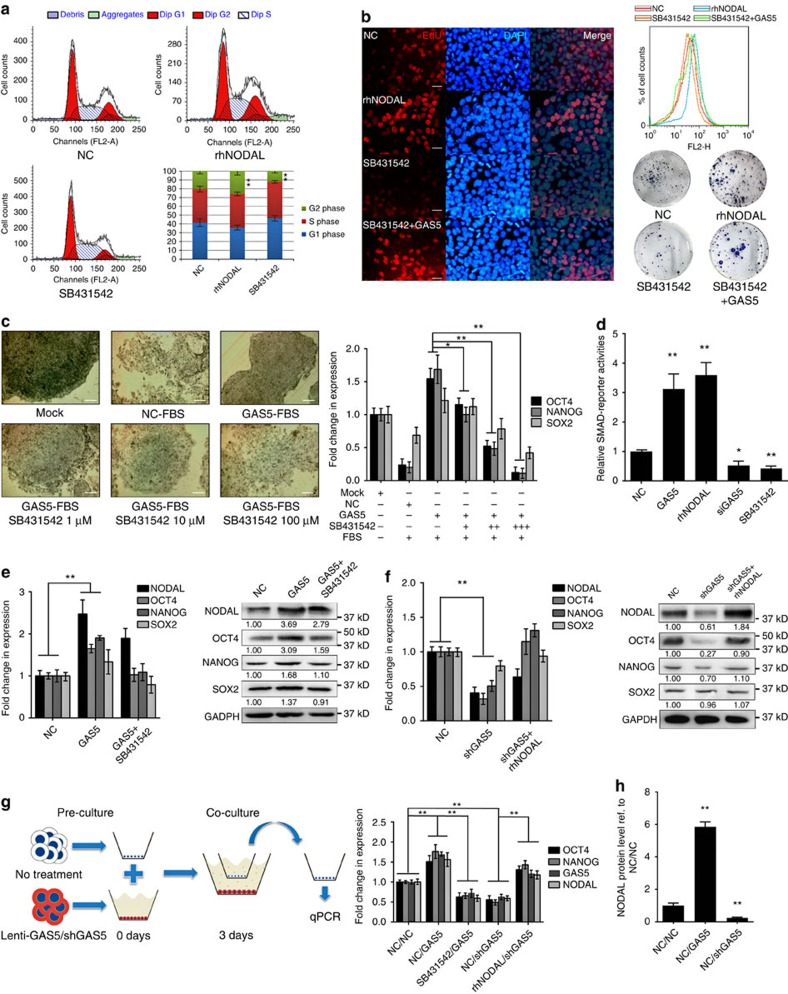
NODAL is required for GAS5 to promote self-renewal in hESCs. (**a**) Cell cycle analysis of recombinant human NODAL (rhNODAL) or SB431542 treatment compared with negative control (NC) in hESCs. DMSO is used as NC. s.d. of three replicates is indicated, ratio of G2 phase were compared for significance, **P<0.01, *t*-test, *n*=3. (**b**) EdU corporation analysis (*n*=3) of rhNODAL or SB431542-treated hESCs compared with control. Quantification of EdU corporations using flow cytometry (*n*=3) were shown in the upper right panel. The lower right panel shows the colony formation assay of hESCs with indicated treatment (*n*=2). Scale bar, 50 μm. (**c**) Fetal bovine serum induced differentiation assay in hESCs. The left panel shows the microscopy images of alkaline phosphatase stained hESCs under indicated treatment. Scale bar, 200 μm. The right panel shows the qPCR analysis of the pluripotency genes in the indicated treatment group. SB431542, an ALK4/7 inhibitor. The significance of changes in OCT4 and NANOG expression were compared with GAS5+FBS group. Here Mock represents untreated hESCs, NC represents FBS-induced hESCs with no other treatment. *P<0.05, **P<0.01, *t*-test, *n*=3. (**d**) Luciferase activities of SMAD reporter in different treatment groups in hESCs. *P<0.05, **P<0.01, *t*-test, *n*=4. (**e**,**f**) Analysis of expression changes of NODAL, OCT4, NANOG and SOX2 in the indicated groups. Westerm Blot analyses are shown in the right panel. NC represents hESCs treated with empty vector transfection. **P<0.01, *t*-test, *n*=3. (**g**) The left panel shows the scheme for co-culture assay. Right panel shows the mRNA level of pluripotency genes and GAS5 of the upper chamber cells in the indicated groups. NC/NC stands for no treatment in both upper and lower chamber, NC/GAS5 stands for GAS5 overexpression in the lower chamber cells, SB431542/GAS5 stands for SB431542 treatment in the chambers and GAS5 overexpression in the lower chamber cells. rhNODAL/shGAS5 stands for rhNODAL treatment in the chambers and GAS5 knockdown in the lower chamber cells. **P<0.01, *t*-test, *n*=3. (**h**) NODAL ELISA testing the level of NODAL secreted in the indicated groups. **P<0.01, *t*-test, *n*=4. See also Supplementary Fig. 4. Error bars represent standard deviation of the indicated experiment replicates. RNA level of β-actin served as internal reference for qPCR.(**a**) Cell cycle analysis of recombinant human NODAL (rhNODAL) or SB431542 treatment compared with negative control (NC) in hESCs. DMSO is used as NC. s.d. of three replicates is indicated, ratio of G2 phase were compared for significance, **P<0.01, *t*-test, *n*=3. (**b**) EdU corporation analysis (*n*=3) of rhNODAL or SB431542-treated hESCs compared with control. Quantification of EdU corporations using flow cytometry (*n*=3) were shown in the upper right panel. The lower right panel shows the colony formation assay of hESCs with indicated treatment (*n*=2). Scale bar represents 50 μm. (**c**) Fetal bovine serum induced differentiation assay in hESCs. The left panel shows the microscopy images of alkaline phosphatase stained hESCs under indicated treatment. Scale bar represents 200 μm. The right panel shows the qPCR analysis of the pluripotency genes in the indicated treatment group. SB431542, an ALK4/7 inhibitor. The significance of changes in OCT4 and NANOG expression were compared with GAS5+FBS group. Here, Mock represents untreated hESCs, NC represents FBS-induced hESCs with no other treatment. *P<0.05, **P<0.01, *t*-test, *n*=3. (**d**) Luciferase activities of SMAD reporter in different treatment groups in hESCs. *P<0.05, **P<0.01, *t*-test, *n*=4. (**e**,**f**) Analysis of expression changes of NODAL, OCT4, NANOG and SOX2 in the indicated groups. Westerm blot analyses are shown in the right panel. NC represents hESCs treated with empty vector transfection. **P<0.01, *t*-test, *n*=3. (**g**) The left panel shows the scheme for co-culture assay. Right panel shows the mRNA level of pluripotency genes and GAS5 of the upper chamber cells in the indicated groups. NC/NC stands for no treatment in both upper and lower chamber, NC/GAS5 stands for GAS5 overexpression in the lower chamber cells, SB431542/GAS5 stands for SB431542 treatment in the chambers and GAS5 overexpression in the lower chamber cells. rhNODAL/shGAS5 stands for rhNODAL treatment in the chambers and GAS5 knockdown in the lower chamber cells. **P<0.01, *t*-test, *n*=3. (**h**) NODAL ELISA testing the level of NODAL secreted in the indicated groups. **P<0.01, *t*-test, *n*=4. See also Supplementary Fig. 4. Error bars represent s.d. of the indicated experiment replicates. RNA level of β-actin served as internal reference for qPCR.

Given the contrasting roles of NODAL in embryonic development, we first tested the contributions of NODAL to hESC self-renewal. We examined the cell cycle and EdU incorporation of hESCs treated with rhNODAL or SB431542, a NODAL receptor inhibitor. Similar to GAS5 overexpression, rhNODAL treatment increased hESC self-renewal, while SB431542 exerted contrasting effects (Fig. 4a,b). The effects of SB431542 were partially rescued by GAS5 transfection in both EdU and colony formation assays (Fig. 4b, lower right panel; Supplementary Fig. 4a). qPCR analysis showed that NODAL increases expression of the pluripotency factors OCT4 and NANOG in a dose-dependent manner (Supplementary Fig. 4b); however, NODAL had little effect on SOX2 expression, which was consistent with of previous findings^28^,^29^.

We next tested these effects in an FBS-induced hESC differentiation model. By adding different doses of SB431542 to GAS5-overexpressing, FBS-induced hESCs, we found that GAS5 overexpression improved AP staining (Fig. 4c) and pluripotency gene expression during differentiation (Supplementary Fig. 4c), which was reversed by NODAL inhibition in a dose-dependent manner (Fig. 4c).

Using a luciferase reporter containing multiple SMAD responsive elements (pGMSMAD-Lu), we confirmed its downstream activation in both GAS5-overexpressing and rhNODAL-treated cells. In contrast, GAS5 knockdown and ALK4/7 inhibition reduced luciferase activity (Fig. 4d). We next tested whether NODAL is essential for GAS5 function. We first tested the effects of SB431542 treatment in GAS5-overexpressing hESCs and found that the messenger RNA (mRNA) and protein levels of the pluripotency factors were decreased, except for those of NODAL (Fig. 4e). In GAS5 knockdown cells, we found that rhNODAL incorporation rescued the expression of OCT4 and NANOG (Fig. 4f). Taken together, these results indicate that GAS5 function is largely dependent on NODAL signalling.

NODAL is an autocrine cytokine. Thus, we tested whether GAS5 affects NODAL secretion in hESCs via co-culture assay. First, we seeded ‘donor' hESCs stably expressing Lenti-GAS5, Lenti-shGAS5 or control virus into the lower chamber and allowed the cells to reach at least 60% confluence to ensure sufficient NODAL secretion. Then, we seeded ‘receiver' hESCs into the upper chamber and co-cultured the cells for an additional 3 days. The E7 medium contained no extra NODAL or Activin (Fig. 4g). qPCR analysis of the ‘receiver' cells showed that co-culture with GAS5-overexpressing cells increased NODAL and pluripotency factor mRNA levels compared with the cells infected with the control vector. ALK receptor inhibition by SB431542 attenuated these effects (Fig. 4g). NODAL protein level elevation in the supernatant was also confirmed by enzyme-linked immunosorbent assay (ELISA; Fig. 4h). Collectively, these data demonstrate that NODAL secretion is required for GAS5 to enhance hESC self-renewal.

### NODAL is post-transcriptionally regulated by GAS5

**Figure 5:**
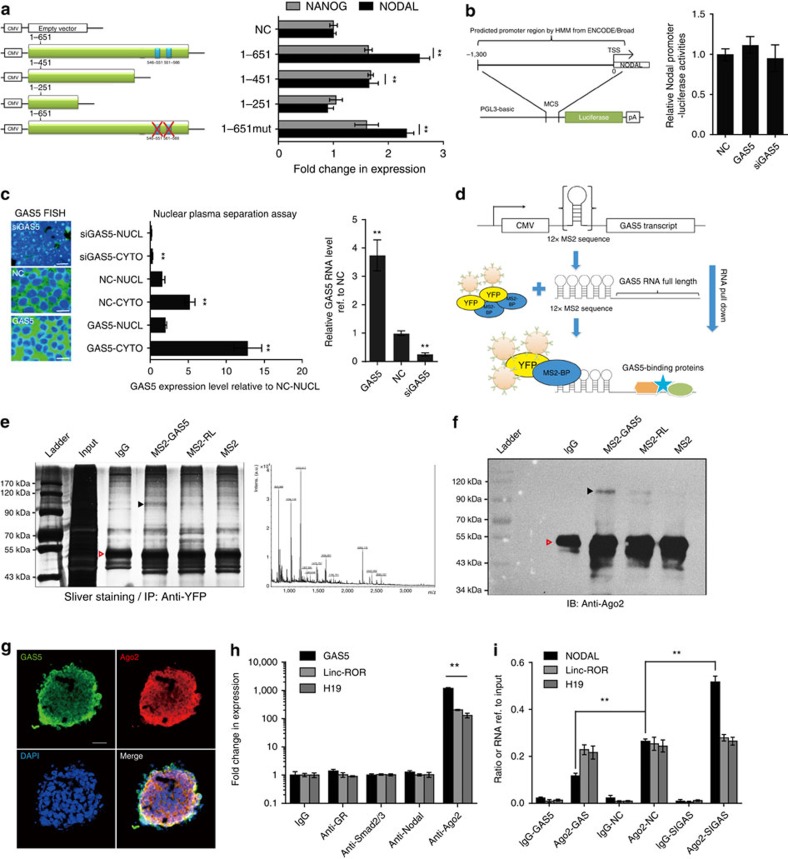
NODAL is post-transcriptionally regulated by GAS5. (**a**) A scheme of vectors expressing different GAS5 truncated transcript. The inserted length of each transcript is indicated and the blue square indicates the position of validated GR-binding sequence. 1-651mut represents the GR-binding sequence-mutated construct. The right panel shows the mRNA level of NANOG and NODAL in different treatment groups. **P<0.01, *t*-test, *n*=3. (**b**) The left panel shows the scheme of constructing *NODAL* promoter reporter. The right panel shows the luciferase activities of *NODAL* promoter reporter in hESCs with indicated treatment. NC represents *NODAL* promoter reporter transfected hESCs treated with empty vector, *t*-test, *n*=3. (**c**) The nuclear-plasma separation assay of GAS5 overexpressed or knockdown hESCs are shown in the middle panel using qPCR. Respective FISH images are shown in the left panel, and the GAS5 expression of analysed using total RNAs are shown in the right panel (*n*=3). NUCL represents nuclear hESC extracts, CYTO represents cytoplasm hESC extracts. Scale bar, 20 μm. All groups were compared with NC or NC-NUCL group respectively. **P<0.01, *t*-test, *n*=3. (**d**) A scheme of MS2-mediated pulldown of GAS5 and its binding protein. (**e**) The silver staining of pull-down products (*n*=2), the red triangle indicates the antibody band, whereas black triangle indicates the bands and position that is cutoff for mass spectrometry analysis shown in the right panel. (**f**) Western blot shows the AGO2 expression in different pulldown groups as indicated by the black triangle (*n*=2). The red triangle indicates the antibody heavy chain bands, which were used in pull-down experiment and react with the secondary antibody. (**g**) Confocal microscopy of AGO2 and GAS5 transcript. Scale bar, 100 μm. (**h**) RNA immune-precipitation analysis of the RNA levels of precipitated GAS5, linc-ROR, H19 using indicated protein antibodies by qPCR. **P<0.01, *t*-test, *n*=3. (**i**) AGO2 competing assay using AGO2 antibody-mediated RNA-IP. RNA levels of precipitated NODAL, linc-ROR, H19 are tested using qPCR. **P<0.01, *t*-test, *n*=2. Error bars represent s.d. of the indicated experiment replicates. RNA level of β-actin served as internal reference for qPCR. See also Supplementary Fig. 5.

Several reports have demonstrated the involvement of GAS5 in the mammalian target of rapamycin (mTOR) and glucocorticoid receptor (GR) pathways^18^,^30^. Therefore, we aimed to determine whether the function of GAS5 in hESC self-renewal is related to these pathways. Because the expression of GAS5 can be elevated by mTOR inhibition^30^, we first tested whether mTOR inhibition by the mTOR inhibitor rapamycin would increase the expression of pluripotency genes. The results showed that pluripotency gene expression and NODAL levels did not significantly decrease until day 3 of treatment (*t*-test Supplementary Fig. 5a), which is inconsistent with the effects of GAS5. GR function can be inhibited by GAS5, so we performed GR knockdown and activation and used dexamethasone, a GR agonist, to analyse the effects. The results showed that neither GR knockdown nor stimulation significantly affected pluripotency gene expression or NODAL expression (*t*-test. Supplementary Fig. 5b,c). In addition, previously identified GR-binding element mutations^18^ in the GAS5 transcript did not prohibit NODAL and NANOG upregulation in GAS5-overexpressing hESCs (Fig. 5a). On the basis of these results, we hypothesized that other GAS5-related mechanisms modulate NODAL expression.

We searched for the functional segment of GAS5 by constructing several vectors encoding different truncated fragments of GAS5 (Fig. 5a, left panel). qPCR results showed that the GAS5 transcript increases NODAL and Nanog expression in a length-dependent manner (Fig. 5a, right panel) and that the segment from 1–251 bp has no function. Next, we investigated the possibility that GAS5 directly regulates *NODAL* transcription. We tested the luciferase activity of the *NODAL* promoter in both hESCs and HEK-293T cells and noted no significant changes in luciferase activity, when GAS5 was overexpressed or silenced (*t*-test Fig. 5b; Supplementary Fig. 5d). We also analysed the asymmetric enhancer region of *Nodal* to determine whether GAS5 promotes NODAL expression by transcriptional regulation (Supplementary Fig. 5e). The results showed that GAS5 modulation does not alter asymmetric enhancer reporter luciferase activity in hESCs. We also assessed GAS5 transcript's localization in either GAS-overexpressing or GAS5 knockdown hESCs and found that the levels of cytoplasmic GAS5 transcripts were significantly affected (*t*-test, *P*<0.01 Fig. 5c). Taken together, we hypothesized that indicating that cytoplasmic GAS5 may play an important role in modulating NODAL expression.

The regulatory mechanisms of lncRNA in the cell cytoplasm involve RNA–RNA or RNA–protein interactions. To examine direct RNA–RNA interactions, we investigated the possibility of sequence homology between the GAS5 and NODAL transcripts and found none (Supplementary Fig. 5f). To examine RNA–protein interactions, we performed an RNA immunoprecipitation (RIP) assay using an MS2-binding protein (MS2bp) system, in which tagged MS2bp specifically binds RNA containing MS2-binding sequences (MS2bs; Fig. 5d). We constructed an MS2bs-GAS5 vector and used MS2bp to pulldown the transcript. SDS–polyacrylamide gel electrophoresis (SDS–PAGE) and silver staining were performed to identify a differential protein band (Fig. 5e, indicated by a solid arrow), and subsequent liquid chromatography–mass spectrometry/mass spectrometry proteomic analyses were performed to analyse the content of this band (Fig. 5e). By analysing these data, we found that AGO2, a key component of the miRNA-mediated silencing complex, is more enriched in MS2-GAS5 pulldown products than other control products (Fig. 5f). However, to exclude other critical proteins that may have been missed by analysing this single differentially stained band, we performed label-free high-throughput proteomics analysis using MS2-GAS5 pulldown products. Gene ontology analysis of all predicted pulldown proteins showed enrichment of translation regulation-related and miRNA-mediated silencing-related proteins (Supplementary Fig. 5g; Supplementary Data 3), supporting the idea that an AGO2- and miRNA-based mechanism may underlie the function of GAS5. RNA FISH analysis combined with immunofluorescence using a specific antibody also showed that GAS5 and AGO2 co-localized in the cytoplasm of hESCs (Fig. 5g) and that GAS5 does not directly bind to its downstream effectors NODAL and SMAD2/3 (Fig. 5h). These data suggest that GAS5 binds to AGO2 and may be associated with miRNA-mediated silencing.

Previous reports have demonstrated that lncRNA and other transcripts serve as ‘sponges' that regulate target transcripts by competing with post-transcriptional elements. We therefore employed AGO2-RIP to test whether differential GAS5 expression affects the binding of AGO2 to NODAL transcripts. The results showed that on GAS5 overexpression, AGO2-NODAL binding was significantly decreased, while on GAS5 silencing, this binding was significantly increased (*t*-test, *P*<0.01 Fig. 5i). These findings suggest that GAS5 regulates NODAL by competing with AGO2 post transcriptionally.

### GAS5-interacting miRNAs regulate NODAL expression

**Figure 6:**
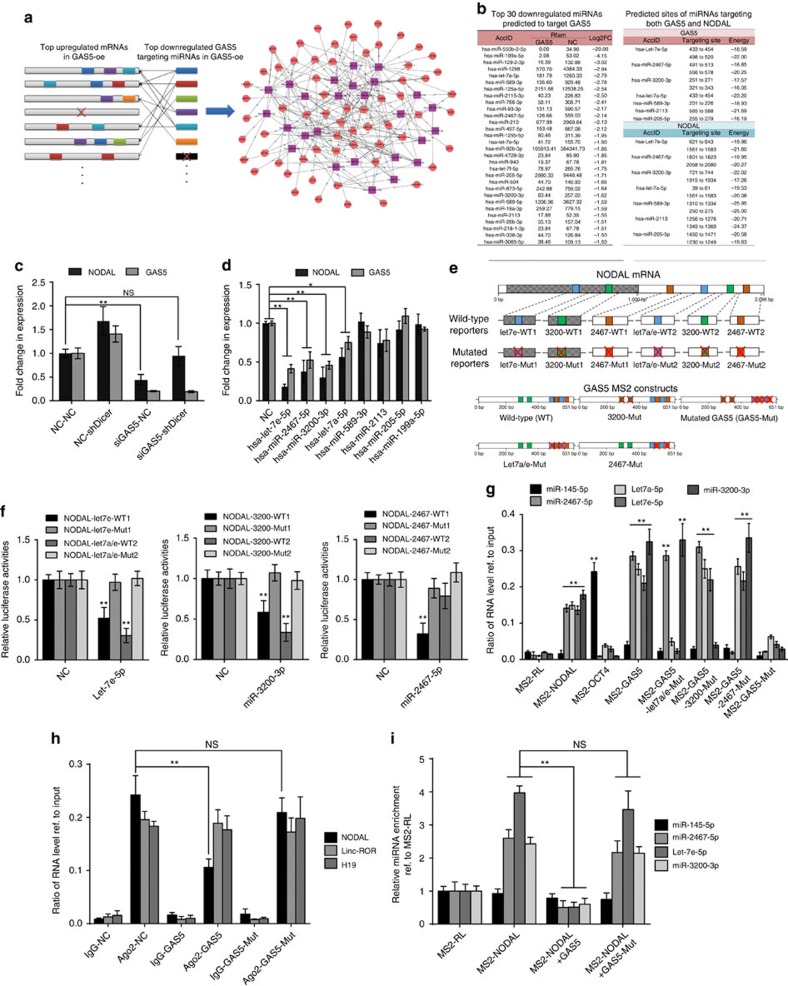
Mirnome analysis revealed that GAS5 competes miR-2467/-3200/Let7e to sustain NODAL expression in hESCs. (**a**) A scheme of miRNA target network showing the relationship of GAS5 downregulated miRNAs and their predicted target mRNAs. (**b**) A list of top 30 downregulated candidate miRNAs on GAS5 overexpression analysed by high-throughput miRNA sequencing. The right panel shows predicted candidate miRNAs that bind to both GAS5 and NODAL. (**c**) The analysis of NODAL and GAS5 expression in Dicer deficient hESCs. ShDicer represents lentiviral-mediated Dicer knockdown. NC represents scramble siRNA control. **P<0.01, *t*-test, *n*=3. (**d**) The effect of candidate miRNAs on the mRNA levels of GAS5 and NODAL in hESCs. MiRNA mimics are transfected into hESCs at the concentration of 20 pmol per ml. **P<0.01, *t*-test, *n*=3. (**e**) A graph illustrating the binding sites of candidate miRNAs to GAS5 and NODAL transcript, and the scheme of constructing wild-type and mutant NODAL luciferase miRNA reporters and GAS5 MS2 constructs. The red cross indicates mutated sites, see also Supplementary Data 4. (**f**) Luciferase activities of relative reporter in **e** with different miRNAs transfection in HEK-293T cells. *P<0.05, **P<0.01, *t*-test, *n*=3. (**g**) qPCR analysis of MS2-mediated candidate miRNAs pulldown assay. Data were normalized to input, and the enrichment ratios compared with input level were shown. MS2-RL serve as a negative control, and MS2-OCT4 are served as positive control in detecting miR-145-5p. **P<0.01, *t*-test, *n*=2. (**h**) AGO2 competing assay using AGO2 antibody-mediated RNA-IP in the indicated groups. RNA levels of precipitated NODAL, linc-ROR and H19 are tested using qPCR. NS, not significant, **P<0.01, *t*-test, *n*=2. (**i**) MS2-mediated miRNA pulldown in the indicated transcript overexpression groups. MS2-RL serves as control. NS, not significant, **P<0.01, *t*-test, *n*=2. Error bars represent s.d. of the indicated experiment replicates. RNA level of β-actin served as internal reference for qPCR. See also Supplementary Fig. 6.

The binding of AGO2 to a specific transcript requires specific miRNA. Therefore, we first used lentivirus-mediated knockdown of Dicer expression and found global miRNA expression reductions in hESCs compared with control cells (Supplementary Fig. 6a). Simultaneously, the GAS5 knockdown induced decreases in NODAL expression were attenuated in Dicer knockdown hESCs (Fig. 6c), which indicated a key role of miRNA-mediated regulation in this process. To identify GAS5- and NODAL-interacting miRNAs, we analysed the miRNome via high-throughput sequencing and constructed a network, in which the abundances of both target genes and miRNAs were considered. To scale down the network, we included only the top 30 miRNAs that were predicted to target GAS5 (Fig. 6a). We found that seven miRNAs in this network also targeted NODAL (Fig. 6b).

To identify the functions of these candidate miRNAs, we transfected their mimics or inhibitors directly and analysed the changes in GAS5 and NODAL expression in hESCs. The results showed that miR-2467-5p, -3200-3p and -let-7a/e-5p overexpression significantly reduced both NODAL and GAS5 RNA levels (*t*-test, *P*<0.01 Fig. 6d; Supplementary Fig. 6b), whereas their inhibition caused RNA upregulation (Supplementary Fig. 6c). Validation of target sites were done using luciferase reporter system, including NODAL wild-type and site-mutated constructs (Fig. 6e; Supplementary Data 4). We found that these miRNAs significantly suppressed the luciferase activities of the wild-type reporters (*t*-test, *P*<0.01), but not those of the site-mutated reporters, except for a reporter featuring mutations at site 2 of miR-2467 in NODAL (Fig. 6f). To validate the direct binding of the predicted miRNAs to GAS5 transcripts, we performed an MS2-mediated RIP analysis using constructs containing either wild-type GAS5 or mutated GAS5 (Fig. 6e) fused with MS2bs. qPCR analysis showed that miR-2467-5p, miR-3200-3p and let-7a/e-5p were significantly enriched in the wild-type GAS5 and NODAL transcripts, and that no significant enrichment occurred in the relative site-mutated GAS5 transcripts or in a GAS5 transcript, in which all miRNA-binding sites were mutated (Fig. 6g). The binding of miR-145-5p to OCT4 3′-untranslated region (UTR) served as a positive control. We again performed an Ago2-mediated pulldown (Fig. 6h) and an MS2-mediated pulldown (Fig. 6i) to determine whether all mutated GAS5 sites still affected NODAL expression. As expected, the mutated GAS5 transcript did not compete with Ago2 to bind the NODAL transcript. These data provide evidence of the direct binding of the candidate miRNAs to GAS5 and NODAL mRNA.

How GAS5 affects these miRNAs remains unknown. Here we first performed northern blotting and confirmed their existence (Supplementary Fig. 6e). Using qPCR analysis, we noted downregulation of the validated miRNAs in conjunction with wild-type GAS5 overexpression, but not GAS5mut overexpression (*t*-test, *P*<0.01 Supplementary Fig. 6f), indicating that miRNA degradation mechanism may be associated with this interaction. To clarify this point, we explored the turnover of these RNAs using actinomycin D, an inhibitor of *de novo* RNA transcription. The turnover of validated miRNAs was accelerated on GAS5 expression (Supplementary Fig. 6g,h). At the same time, overexpression of miR-2467-5p, miR-3200-3p or let-7a/e-5p also promoted GAS5 turnover (Supplementary Fig. 6i). These data suggest that the GAS5 transcript post-transcriptionally regulates the levels of miR-2467-5p, miR-3200-3p and let-7a/e-5p, and promotes their degradation.

### MiR-2467/3200/Let7e are essential for GAS5 function

**Figure 7:**
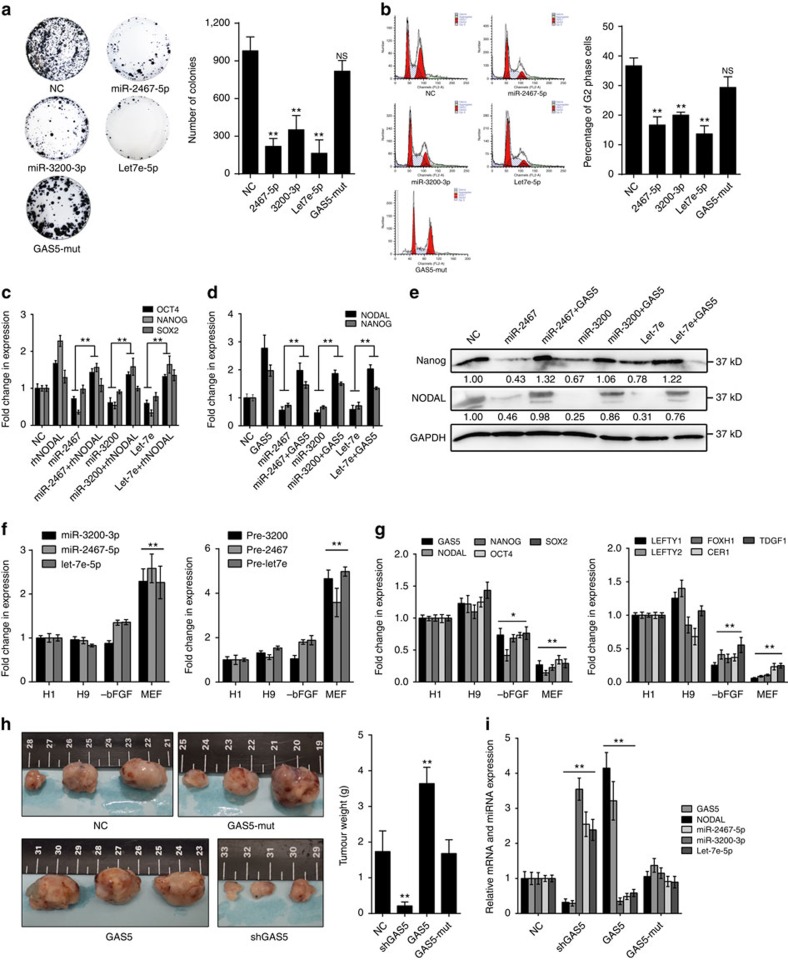
MiR-2467/3200/Let7e inhibit self-renewal and attenuate GAS5-mediated NODAL signalling in hESCs. (**a**) Colony formation assay with different miRNA transfection in hESCs. Quantification of colonies are shown in the right panel. NS, not significant; NC, microRNA mimic control overexpression group. **P<0.01, *t*-test, *n*=3. (**b**) Cell cycle analysis of different miRNA overexpression in hESCs. Comparison of cell ratios in G2 phase are shown in the right panel. NS, not significant; NC, microRNA mimic control overexpression group. **P<0.01, *t*-test, *n*=3. (**c**–**e**) The mRNA level of pluripotency genes in miRNA-transfected cells combined with rhNODAL incorporation (**c**) or GAS5 overexpression (**d**). The relative western blot results are shown in the right panel (**e**). NC, microRNA mimic control overexpression group. **P<0.01, *t*-test, *n*=3. (**f**) Analysis of mature microRNA and pre-miRNA expressions between different differentiation methods in H1 hESCs. **P<0.01, *t*-test t, *n*=3. (**g**) The analysis of expression changes of pluripotency genes and NODAL signalling-related genes during different differentiation of H1 hESCs. *P<0.01, **P<0.01, *t*-test, *n*=3. Here, −bFGF represents bFGF-deprived hESC medium; MEF represents mouse embryonic fibroblasts culture medium (which contains fetal bovine serum). (**h**) Teratoma formation assay using lentiviral stably transfected hESCs injected into the back cutaneous of NOD/SCID mice. Six weeks after the injection, tumours were dissected, photographed (left panel) and weighed (right panel). The ruler scales are 0.5 mm per minor mark. **P<0.01, *t*-test, *n*=3. (**i**) Levels of GAS5, NODAL, miR-2467, miR-3200 and Let7e in the tumours were evaluated using qPCR. **P<0.01, *t*-test, *n*=3. Error bars represent s.d. of the indicated experiment replicates. RNA level of β-actin served as internal reference for qPCR. See also Supplementary Fig. 7.

To investigate the functions of these miRNAs in hESC self-renewal, we employed colony formation assays and cell cycle analysis. The results showed that overexpression of these miRNAs significantly disrupted hESC self-renewal (Fig. 7a,b). Increased expression of the pluripotency factors OCT4 and NANOG, but not SOX2, was facilitated via inhibition of these miRNAs (Supplementary Fig. 7a). Unlike the wild-type transcript, the GAS5-mut transcript exerted no effects (Fig. 7a,b; Supplementary Fig. 7b). We found that miR-2467-5p, miR-3200-3p and let-7a/e-5p did not directly target these pluripotency factors, using dual luciferase assays (Supplementary Fig. 7c).

Because miR-2467-5p, miR-3200-3p and let-7a/e-5p facilitated expression of similar phenotypes resembling NODAL inhibition, we investigated whether NODAL rescues the effects of these miRNAs. We found that rhNODAL rescues pluripotency gene downregulation caused by individual miRNA overexpression (Fig. 7c), suggesting that NODAL is the critical target of these miRNAs in hESCs. To determine whether these miRNAs are essential for GAS5-mediated NODAL expression, we transfected these miRNAs mimics into GAS5-overexpressing hESCs, and found that both pluripotency gene expression and NODAL expression were attenuated compared with control cells (Fig. 7d). Western blot analysis confirmed this finding (Fig. 7e). On the basis of these data, we concluded that miR-2467-5p, miR-3200-3p and let-7a/e-5p play important roles in GAS5-mediated NODAL regulation.

The biological significance of miR-2467-5p, miR-3200-3p, let-7a/e-5p and their regulatory transcripts is unknown. Therefore, we detected the changes in their endogenous expression during hESC differentiation. Real-time analysis of both undifferentiated (H1 and H9) and differentiated depletion of basic fibroblast growth factor (−bFGF) and mouse embryonic fibroblasts (MEF) induction) cells showed that miR-2467-5p, miR-3200-3p and let-7a/e-5p expression was significantly upregulated during differentiation (*t*-test, *P*<0.01 Fig. 7f), accompanied by gradual reductions in GAS5, NODAL signalling-related gene and pluripotency gene expression (Fig. 7g), indicating the existence of an inverse relationship between the expression of these miRNAs and GAS5-mediated NODAL signalling.

We validated the function and mechanism of GAS5 in hESCs and demonstrated the existence of a regulatory loop, in which pluripotency-driven GAS5 promoted NODAL signalling by competing with NODAL-targeting miRNAs. However, to gain additional *in vivo* evidence, we performed teratoma formation analysis. By injecting stably transfected hESCs into the backs of nude mice, we found that GAS5-overexpressing hESCs formed larger teratomas than control cells, while mutant GAS5-overexpressng cells did not form significantly larger tumours. GAS5 knockdown resulted in reduced tumour size (Fig. 7h). We also found that GAS5 increased NODAL expression, while reducing the expression of its targeting miRNAs in these tumours (Fig. 7i).

### Specific interactomes are essential for GAS5 function

**Figure 8:**
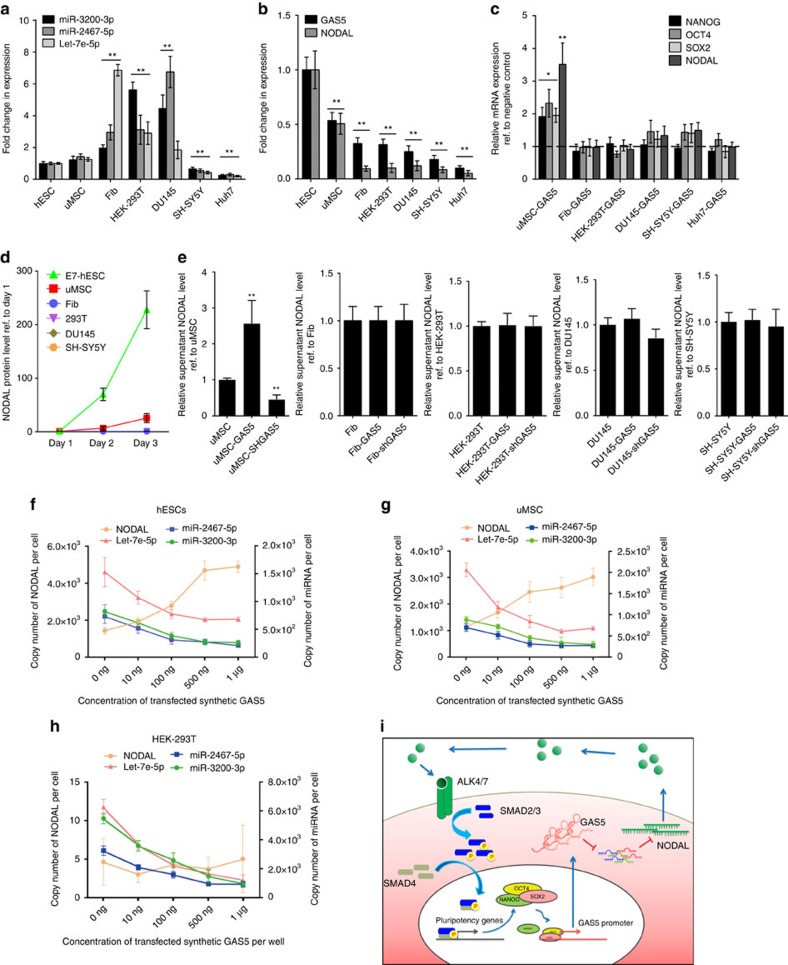
Specific interactome is essential for GAS5 to mediate NODAL signalling. (**a**,**b**) The analysis of the expression level of NODAL, GAS5 (**b**) and their interacting miRNAs (**a**) in different cell types. **P<0.01, *t*-test, *n*=3. (**c**) The relative mRNA level of pluripotency genes and NODAL in the GAS5 overexpressed groups were compared with different cell types using qPCR. The expression levels of pluripotency genes and NODAL in each cell type is compared with their relative empty vector expressed group, which were shown as a dashed line. **P<0.01, *t*-test, *n*=3. (**d**,**e**) ELISA analysis of the secreted NODAL in the cell culture medium of different cell types (**d**) and GAS5 overexpressed or knockdown cells (**e**). All cells for supernatant collecting were cultured using serum-free medium. **P<0.01, *t*-test, *n*=3. (**f**–**h**) Standardized quantitative PCR detecting the expression change of indicated miRNA and NODAL transcript in hESCs (**f**), uMSCs (**g**) and HEK-293T cells (**h**) under different concentration of synthetic full-length GAS5 RNA transfection. *n*=3. (**i**) The graphical summary of the function and mechanism of GAS5. Error bars represent s.d. of the indicated experiment replicates. RNA level of β-actin served as internal reference for qPCR. See also Supplementary Fig. 8.

To explore the function of GAS5 in other cells, we first evaluated the expression levels of the above miRNAs and NODAL in other cell lines, and found that although these interacting miRNAs are differentially expressed in different cell lines (Fig. 8a), the expression of NODAL was consistently lower in other cell lines (Fig. 8b). Furthermore, we found that GAS5 did not promote pluripotency and NODAL expression in cells other than hESCs and umbilical cord mesenchymal stem cells (uMSCs) (Fig. 8d).

As previous studies have shown that NODAL is not expressed in normal tissues^28^ and that specific ratios of competing endogenous RNA (ceRNA)/target RNA are critical for the formation of functional competing endogenous pairs^31^,^32^, we hypothesized that lack of NODAL expression may be the cause of the loss of GAS5 function in other cells. To verify this hypothesis, we evaluated NODAL secretion levels in these cell types and found that only hESCs and uMSCs secrete detectable levels of NODAL (Fig. 8d), and that only in uMSCs does GAS5 induce NODAL section (Fig. 8e). We measured the copy number changes of these miRNAs and NODAL after transfecting different cells with different concentrations of *in vitro*-synthesized full-length GAS5 transcripts. The results showed that NODAL expression is initially upregulated after transfection and increases in the presence of higher GAS5 concentrations, whereas the expression of their interacting miRNAs decreases continuously (Fig. 8f). Similar expression patterns were observed in uMSCs (Fig. 8g). Although miRNA expression was gradually downregulated, NODAL expression did not change due to its low or non-existent basal expression level in HEK-293T cells (Fig. 8h), which supported our hypothesis. Here we demonstrated that a cell-specific GAS5 regulation circuit fine tunes NODAL expression via interacting miRNAs, thus promoting hESC self-renewal and pluripotency (Fig. 8i).

## Discussion

LncRNAs are newly identified players in pluripotency and self-renewal regulation in hESCs. Many studies have examined the functions of long ncRNAs in both ESC lineage commitment and the self-renewal^12^,^33^. GAS5 was identified decades ago^34^; however, its function and working mechanism were not identified until recently in tumour suppression^21^,^35^,^36^,^37^,^38^, growth arrest^18^,^22^,^38^,^39^ and affected by rapamycin^40^,^41^. Our finding that GAS5 is highly expressed in hESCs appears to be arbitrary given its significant effect on tumour suppression and proliferation inhibition. However, its role in development was indicated decades ago^16^,^17^,^34^,^39^; among the GAS gene family members, GAS5^42^ and GAS6^17^ were found to be upregulated in ESCs, and only GAS5^42^ was highly expressed in hESCs. Although previous studies have suggested a relationship between GAS5 and hESC self-renewal maintenance, no experiments regarding the function of GAS5 in hESCs have been conducted. To address this idea, we investigated GAS5 expression in hESCs and found that GAS5 maintains hESC pluripotency and self-renewal by promoting NODAL signalling. Because previous reports showed that GAS5 promoted apoptosis, and growth inhibition of various tumour cells^18^,^38^,^43^,^44^ and normal cells^40^,^45^, it is interesting that GAS5 also promotes hESC proliferation, which may due to its modulation to NODAL signalling.

In identifying the signalling pathways that control ESC pluripotency, the members of the TGFβ superfamily were crucial for maintenance of the undifferentiated state^2^,^46^. Unlike mESCs, NODAL is important for hESC self-renewal^47^. NODAL is highly expressed in hESCs and is rapidly downregulated during differentiation^48^. Its overexpression inhibits hESC differentiation into neuro-ectoderm and maintains pluripotency markers^49^. Previous reports^29^,^50^,^51^ have also shown that the NODAL signalling pathway directly controls NANOG expression in hESCs and mouse epiblast stem cells (mEpiSCs), thereby affecting hESC pluripotency and self-renewal. Intriguingly, an important feature of the NODAL pathway is its complete lack of activity in normal tissues^52^, making its role in vertebrate developmental biology more mysterious. In our study, we showed that GAS5 regulates NODAL expression through a competing endogenous network controlled by miRNAs. However, we also tested this interaction in other cell types and did not observe elevated NODAL expression on GAS5 overexpression, suggesting that GAS5 functions differently in other cell types than in hESCs. This phenomenon may be partly explained by the complete lack of NODAL expression in differentiated cells; accordingly, we did not detect NODAL secretion in the supernatants of these cells.

Another possible reason for the unique functions of GAS5 may also be tied to its involvement in competing endogenous networks. Highly expressed site-containing RNAs, whether naturally occurring or delivered as research reagents, can act as ‘sponges' to titrate miRNAs away from other normal targets^53^,^54^,^55^,^56^,^57^. Although many studies have identified competing endogenous networks in various cell types, controversy remains regarding the biological significance of these networks in primary cells^32^. A major concern is the quantity of these ‘sponges' in competing endogenous pairs. However, global RNA sequencing showed that GAS5 is the most abundant lncRNA and it is among the top 50 expressed RNAs in hESCs, with an RPKM (reads per kilobase of transcript per million reads mapped) similar to that of OCT4, which is the most highly expressed pluripotency gene in hESCs. In contrast, NODAL expression is 20 times lower than that of GAS5, and all known interacting miRNAs have copy numbers that are higher than NODAL, but lower than GAS5. Our findings indicate that GAS5-mediated promotion of hESC self-renewal via NODAL expression elevation may depend on certain miRNomes or target ceRNA expression to form this regulatory loop. However, confirming this hypothesis may require additional studies comparing miRNome and transcriptome changes in various cell types.

Here we found that GAS5 not only sponges, but also downregulates and increases the degradation of its target miRNAs. This could be an intriguing issue concerning ceRNA or may be all miRNA-related mechanism. Traditionally, binding of miRNAs to target sites causes translational repression or RNA degradation. Perfect matches appear more likely to cause RNA degradation, and seed region matches appear more likely to cause translational repression. However, such criteria are also changing quickly that can be inferred from previous reports^58^,^59^, for miRNA causes deadenylation and decapping of the target RNA even in an imperfect binding motif. So it is better to understand that miRNA's binding to target RNA could cause not only the degradation of target, but also the instability of miRNA itself^60^,^61^,^62^, which indicates a mutual degradation mechanism exists. Nevertheless, such mechanism is still controversial and needs further investigation to fully reveal this phenomenon.

Since miRNAs are critical in forming ceRNA network, it is hard to fully classify and standardize ceRNA due to the diverse functions of miRNAs. However, whether miRNA and its target degrade or not is certainly not a required factor in defining ceRNA. The fundamental factor in forming a ceRNA pair is that both RNAs share mutual miRNA and one can affect the expression of other target. Since the quantity strongly affects the possible function of ceRNA, it is accepted that ceRNA must have greater expression than the miRNAs and the other functional targets. Still, to make certain such regulation is caused by ceRNA mechanism, one must exclude other possibilities. Our proposed GAS5-miRNA-NODAL model fits these criteria for both GAS5 and NODAL is targeted by these miRNAs, we found and GAS5 is much higher in expression than the others. However, to fully reveal the whole ceRNA network in ESCs and to further define the underling mechanism in such mutual degradation warrant further study.

## Methods

### Informed consent

This study was approved by the Ethics Committee of Second Military Medical University. All embryos were obtained with written informed consent signed by the donors voluntarily for research on human early embryonic development mechanisms with no financial payment.

### Cell culture and embryo collection

Human ESC lines H1, H9 (purchased from WiCell Research Institute, ID: WAe001-A for H1 and WAe009-A for H9) and X-01 (ref. 63); provided by Professor Lei Xiao from Shanghai Institutes for Biological Sciences, ID: SDSHES-01) were routinely maintained on feeder-free cultures with commercial available maintaining medium as instructed. E8, E7 or mTeSR1 (Stemcell Technologies) medium were used for feeder-free cultures as indicated respectively with daily change. Cells were passaged using Gentle Cell Dissociation Reagent (Stemcell Technologies) when 70% clonal confluence was reached. Differentiation by forming EB suspension was carried out in a low attachment six-well plate (Corning) with hESCs medium. Undirected differentiation of hESCs was performed in a feeder-free condition using DMEM supplied with 10% fFBS, 1 mM glutamine, 1% nonessential amino acids (all form Invitrogen) and 0.1 mM β-mercaptoethanol (Sigma-Aldrich). Another alternative differentiation method of feeder-free hESCs involved the use of conditioned hESC medium deprived of bFGF. Cells were routinely tested for mycoplasma contamination, and no contamination was found during the experiments.

Embryos were donated for this study with the informed consent of healthy couples, who already had a healthy baby with no genetic disorders from the same *in vitro* fertilization cycle and wished to donate the remaining cryopreserved embryos. The cleavage stage embryos were collected using fine needle aspiration after fertilization and were stored in liquid nitrogen storage tank. On usage, the embryos were thawed rapidly by taking straws from the liquid nitrogen storage tank, and embryos of different stages were randomly collected for further analysis by the embryologists at the clinical *in vitro* fertilization lab of the Center for Reproductive Medicine, Renai Hospital, Shanghai Jiao Tong University. All human-related procedures were carried out in accordance with guidelines approved by the Ethics Committee for Research on Human Subjects of Second Military Medical University. Samples were used and reported anonymously with the experimental findings.

### Immunofluorescence and FISH

Samples were fixed with 4% paraformaldehyde. For the detection of proteins, samples were blocked with 5% bovine serum albumin, than incubated with rabbit polyclonal anti-Ago2 (1:100, ab32381, Abcam). For the detection of GAS5 and DANCR, samples were further hybridized with commercial available SuperHyb Solution (Tiandz Inc. No.130906-10) containing respective RNA probes. RNA probes were used and labelled with digoxigenin (DIG)-UTP (Roche) using the mMESSAGE T7 Ultra *In Vitro* Transcription Kit (Ambion) in accordance with the manufacturer's directions.

### Western blots

Cells or tissues were resuspended in ice-cold cell lysis buffer (Cell Signalling Technology) with protease inhibitor cocktail (complete mini-tablet, Roche) and incubated for 15–30 min on ice. SDS–PAGE was done in 4–20% Tris-Glycine Gels (Invitrogen) and transferred to platelet-derived growth factor membranes (Millipore). The membranes were blocked in Tris-buffered saline with 0.05% Tween-20 and 5% milk, incubated with primary and secondary antibodies, and washed according to standard procedures. Primary antibodies were rabbit polyclonal anti-Nodal (1:1000, ab109317, Abcam), rabbit polyclonal anti-Oct4 (1:1000, ab19857, Abcam), rabbit polyclonal anti-Nanog (1:1000, ab21624, Abcam), rabbit polyclonal anti-Sox2 (1:1000, ab97959, Abcam), rabbit polyclonal anti-Dicer (1:1000, 20567-1-AP, Proteintech), rabbit polyclonal anti-Ago2 (1:1000, ab32381, Abcam), rabbit monoclonal anti-SMAD2/3 (1:500, #8685, Cell signalling Technology), rabbit polyclonal anti-pSMAD2 (1:500, #3104, Cell signalling Technology), rabbit monoclonal anti-pSMAD3 (1:500, #9520, Cell signalling Technology), rabbit polyclonal anti-GR (1:1000, 24050-1-AP, Proteintech), rabbit polyclonal anti-Histone (1:10000, 15446-1-AP, Proteintech) and rabbit polyclonal anti-GAPDH (1:1000, 10494-1-AP, Proteintech). Uncropped images were shown in Supplementary Fig. 9.

### Lentiviral transduction and selection in hESCs

Lentivirus production and tittering were done by GenePharma Corp, all lentiviruses contain a puromycin selection tag. For lentivirus transduction in hESCs, the lentiviruses were added the second day of hESC passage at multiplicity of infection 10, and continued to be added in the medium with daily change for another 2 days. To generate stable colonies, puromycin was added on the fifth day of passage at the concentration of 100 ng ml^−1^ for selection, and hESCs colonies that survived were passaged and analysed by either quantitative PCR with reverse transcription (qRT–PCR) or fluorescence microscopy.

### Vector construction and small RNA synthesis

The complementary DNA (cDNA) encoding lncRNA-GAS5, LOC100506647, LINC00458 or lncRNA-DANCR was PCR-amplified by the Pfu Ultra II Fusion HS DNA Polymerase (Stratagene) and subcloned into the XhoI and EcoRI sites of pcDNA3.1 vector (Invitrogen). The pcDNA3.1-GAS5 with point mutations in the DBD mimic sequence, miR-2467, miR-3200 and let-7a/e response elements was synthesized using a QuikChange Site-Directed Mutagenesis kit (Stratagene). Vector pcDNA3.0-MS2 (12X) was double digested with NotI and XhoI, and the GAS5 wild-type and mutated fragment was synthesized and subcloned into this construct. The3-′UTR of NODAL were amplified using PCR and subcloned into the pMir-Report vector (Promega) for luciferase reporter assay using the one-step-directed cloning kit (Novoprotein, Shanghai, China). The 3′-UTR of NODAL mRNA containing the miRNA-binding sites were mutated and subcloned into pMir-Report.

SiRNAs, miRNA mimics, miRNA inhibitors and other oligoes used in vector construction were all designed and synthesized by GenePharma Corp. RNAiMAX (Invitrogen) was used for small RNA transfection. SMAD luciferase reporter (pGMSMAD-Lu) was purchased from Genomeditech (USA) where multiple SMAD responsive element was inserted before the luciferase gene.

### Luciferase reporter transfection and dual luciferase assay

Luciferase reporter transfection and dual luciferase assay was performed as following. In brief, HEK-293T cells were plated in 96-well plates and transfected using Lipofectamine 2000 (Invitrogen) with 50 ng pMir-Report vector (carrying firefly luciferase, Promega) inserted with indicated target sequence, empty vector was served as a control, and a PRL-TK vector (carrying Renilla luciferase, Promega) was transfected simultaneously to serve as internal control. Twenty four hours after transfection, cells were lysed and subjected to luciferase assay according to the manufacturer's protocol. For luciferase reporter assay in hESCs, cells were seeded in 48 well, while other procedures were accordingly. The details of construction of reporter vectors and transfections are shown in the Supplementary Experimental Procedures.

### ChIP and RIP assay

ChIP assays were performed in accordance with the manufacturer's instructions of the EZ-Magna ChIP A/G Kit (Millipore, Billerica, MA, USA). The MS2bp/MS2bs-based RIP assay was performed using the EZ-Magna RIP Kit (Millipore) in accordance with the manufacturer's instructions. MS2bp/MS2bs transfected hESCs were collected and lysed using the kit and an anti-GFP antibody (5 μg per assay, Abcam, # ab290) were ligated to the magnetic beads for RNA pulldown.

### FACS and cell cycle analysis

FACS and flow cytometry analysis were performed according to the standard protocol. For cell cycle analysis, each 1 ml of cell suspension (1–5 × 10^5^ cells) was incubated with the (propidium iodide (PI), Cell Signalling Technology). For EdU analysis, 1–5 × 10^5^ cells from each sample were processed with EdU (EdU Detection Kit, Ribobio), in accordance with the manufacturer's instructions.

### Cell cycle synchronization assay

The cells were treated with NOCODAZOL (Beyotime, Shanghai, China) at concentration of 100 ng ml^−1^ for 12 h when 80% confluent reached. Two hours before experiment, the cells are washed in phosphate-buffered saline for two times and changed for new medium to release the NOCODAZOL. Cells were collected at different time points and fixed according to standard protocol^64^. Cell cycle were analysed using BD FACs-Calibur (BD Biosciences).

### Total RNA isolation and RT–qPCR

Total RNAs from cultured cell lines were extracted with Trizol (Invitrogen). For RT–PCR, after treatment with DNase I (Ambion, DNA-free kit), the complementary DNA was transcribed with Revertra Ace (TOYOBO). For qPCR, the relative expression of different sets of genes was quantified to GAPDH mRNA.

### Northern blotting

Northern blots were performed with DIG-labelled anti-sense probes (DIG Northern Starter Kit, Roche). In brief, total RNA isolated from various cells using Trizol (Invitrogen) was separated by 12% denaturing RNA PAGE and transferred to a positively charged nylon membrane (GE Healthcare) by wet electro-transfer blotting. Membranes were crosslinked by ultraviolet for 3 min, prehybridized for 1 h at 55 °C and hybridized overnight at 60 °C with GAS5 probes complementary to GAS5. After hybridization, membranes were washed twice 10 min with 2 × saline-sodium citrate buffer (SSC) and once 10 min with 0.2 × SSC. AP conjugated anti-DIG antibody is used for secondary labelling. CDP-STAR (Roche) was used for exposure using an Illuminator Chemiluminescent Detection System (Stratagene). For miRNA detection, total miRNAs were extracted using miRcute miRNA extraction kit (Tiangen), *in vitro*-transcribed anti-sense miRNA probes were generated following instruction, the hybridization temperature was adjusted to 55 °C overnight. Uncropped images were shown in Supplementary Fig. 9.

### NODAL ELISA

NODAL levels in the normal culture medium collected for 24 h from different treatment groups were detected with the Human NODAL ELISA Kit (Cusabio, Wuhan, China) according to the manufacturer's instructions. The ELISA kit is tested using the recombinant full-length human NODAL (Abnova, CA, USA) to assess the reliability of the kit.

### Teratoma formation assay

All procedures relating to animal subjects were performed under ARRIVE guideline and institutionally approved protocol deemed in accordance with the guidelines of the Institute of Laboratory Animal Resources, the Second Military Medicine University. Twelve 6-week-old male NOD/SCID mice were obtained from Laboratory Animal Resources. All animals were housed in a specific pathogen-free environment with 12-h photoperiods and ad libitum access to standard chow and water. *In vivo* pluripotency of the treated hESCs were tested as previously described^46^. In brief, H1 hESCs were collected with collagenase IV and were implanted underneath the back skin of the mice. The mice were randomly divided into each group without blinding to receive implantation. Teratoma growth was determined by weekly observation and palpation. Mice were sacrificed killed 6 weeks after implantation. Teratomas were washed weighed and photographed, than part of the tumour were minced for RNA extraction while other part were preserved for future use. The size of the tumours generated in this experiment were within the limit of those allowed in the ethical guidelines of our institution. A standard ruler was used to define the size of the teratomas (0.5 mm per minor mark).

### High-throughput RNA-sequencing and computational analysis

For RNA sequencing, we aligned 100-bp paired-end sequencing reads to the human genome (hg19) using Tophat2/Bowtie2 allowing for five mismatches. We identified read-pair mappings to gene structures derived from RefSeq using the summarize Overlaps function with mode Intersect Strict (Genomic Ranges, Bioconductor). Using these raw counts, we identified genes expressed across the sample groups. We removed genes with a count <10 across all samples prior to statistical analysis. The differential analysis was carried out using edgeR, applying TMM (trimmed Mean of M-values) library normalization and a 0.001 false discovery rate to select expressed transcripts. In addition, we calculated gene-level RPKM values for the same gene set using Cufflinks.

The likelihood of the binding of a mature miRNA to a certain gene's mRNA was evaluated using the miRanda miRNA Target Detection Software (August 2010 Release, http://www.microrna.org/microrna/getDownloads.do).

We used GSEA v2.0 to perform GSEA on various gene signatures. Gene sets were either obtained from the MSigDB database v4.0 or from published gene signatures. Statistical significance was assessed by comparing the enrichment score to enrichment results generated from 1,000 random permutations of the gene set to obtain *P* values (nominal *P* value).

For miRNA: mRNA interacting network construction, we first ranked the top upregulated mRNAs and top downregulated miRNAs after GAS5 overexpression, and excluded low expression mRNAs (<25 normalized counts) and miRNAs (<25 normalized counts). MiRanda algorithm was used to predict miRNA-binding sites and only miRNAs that binds to GAS5 transcript were selected to form the network.

### Isolation of cytoplasm and nuclear RNA

Cytoplasmic and nuclear RNA were isolated and purified using the PARIS Kit (Ambion, CA, USA) according to the manufacturer's instructions. Extracted RNAs were put to reverse transcription immediately for qPCR analysis.

### Statistical analysis

Each experiment was repeated two or three times or more as mentioned in each figure legend. Data are presented as mean±s.d., unless stated otherwise. Student's *t*-test (unpaired, two tailed) was used to compare two groups for independent samples. *P*<0.05 was taken to indicate statistical significance. No statistical method was used to predetermine sample size for these experiments. All observed data were included without exclusion criteria. Data collection was conducted randomly. Uncropped scans of the most important blots are shown in Supplementary Fig. 8b in the Supplementary Information Section, all oligos used in the article are listed in the Supplementary Data 5.

### Data availability

Data for mRNA expression of either GAS5 overexpressed, knockdown or NC hESCs, and miRNA expression of GAS5 overexpressed or NC hESCs have been deposited in the Gene Expression Omnibus database under accession code GSE66993. The authors declare that all data supporting the findings of this study are available within the article and its Supplementary Information Files or from the corresponding author on reasonable request.

## Additional information

**How to cite this article:** Xu, C. *et al*. Long non-coding RNA GAS5 controls human embryonic stem cell self-renewal by maintaining NODAL signalling. *Nat. Commun.* **7,** 13287 doi: 10.1038/ncomms13287 (2016).

**Publisher's note:** Springer Nature remains neutral with regard to jurisdictional claims in published maps and institutional affiliations.

## Supplementary Material

Supplementary Information Supplementary Figures 1-9

Supplementary Data 1 The predicted results of binding of OCT4 and SOX2 to the GAS5 promoter region by JASPAR database

Supplementary Data 2 GO analysis data

Supplementary Data 3 Mass Spectrum results of GAS5-pull down protein fragments

Supplementary Data 4 The targeting motif of GAS5 and NODAL targeted miRNAs

Supplementary Data 5 Oligonucleotide Sequences used in this study
