# Supplementary material for: Long non-coding RNA GAS5 controls human embryonic stem cell self-renewal by maintaining NODAL signalling
Source: Nat Commun. 2016 Nov 4;7:13287. doi: 10.1038/ncomms13287 (PMC5097163; doi:10.1038/ncomms13287)
Supplement: Supplementary Information — Supplementary Figures 1-9 [file ncomms13287-s1.pdf]

Supplementary Figure 1. Houqi

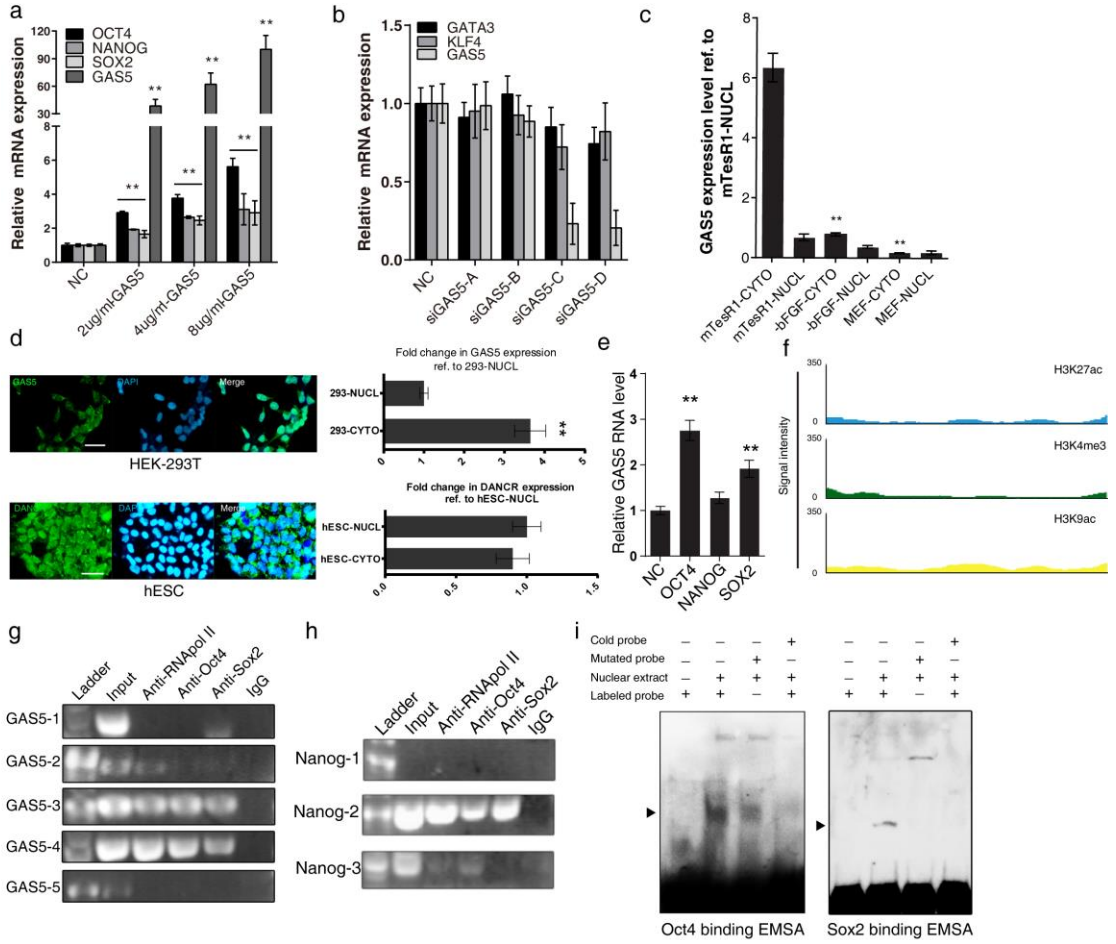

Supplementary Figure 1, related to Figure 1. GAS5 is highly expressed in the cytoplasm of hESCs, and positively correlates with pluripotency.

(**a**) Transfection of different concentration of GAS5-overexpressing plasmids to hESCs. The mRNA level of pluripotency gene GAS5, Oct4, Nanog and Sox2 are detected using qPCR. The concentration indicated represents the amount of plasmids transfected into each 6-well. $^{**}P<0.01$, t-test, n=3. (**b**) Testing the specificity of GAS5 knockdown using the indicated siRNA. KLF4 and GATA3 were predicted to bind none of the siRNA. $^{**}P<0.01$, t-test, n=3. (**c**) The relative GAS5 RNA level in the cytoplasm and nuclear assessed by qPCR. MTesR1 medium represents undifferentiated state, while –bFGF and MEF medium were used to generate differentiation of hESCs. $^{**}P<0.01$, t-test, n=3. (**d**) FISH analysis of GAS5 in HEK-293T cells and lncRNA DANCR (validated nuclear enriched) in hESCs (n=2). The analysis of relative transcript in the subcellular level using nuclear-plasma separation were shown in the right panel. The scale bar represents 100nm. $^{**}P<0.01$,

t-test, n=2. (**e**) The effect of pluripotency genes' overexpression on GAS5 RNA level. $^{**}P$<0.01, t-test, n=3. (**f**) The inactive histone modification state of the predicted *Gas5* promoter region using the ENCODE Ch-IP sequencing data. (**g**) The gel electrophoresis image of ch-IP assay in Fig. 1i. (**h**) The gel electrophoresis image of Ch-IP analysis of the binding of OCT4 and SOX2 to *Nanog* promoter, which served as a positive control for Ch-IP results presented in Fig.1i. (**i**) EMSA of OCT4 (left panel) and SOX2 (right panel) binding using the probe indicated in Fig. 1h (n=2). The probe designed in the GAS5-3 region was used to detect the binding of SOX2, and probe designed in the GAS5-4 region was used to detect the binding of OCT4 (related to Fig. 1h). Error bars represent standard deviation of the indicated experiment replicates. RNA level of β-actin served as internal reference for qPCR.

Supplementary Figure 2. Houqi

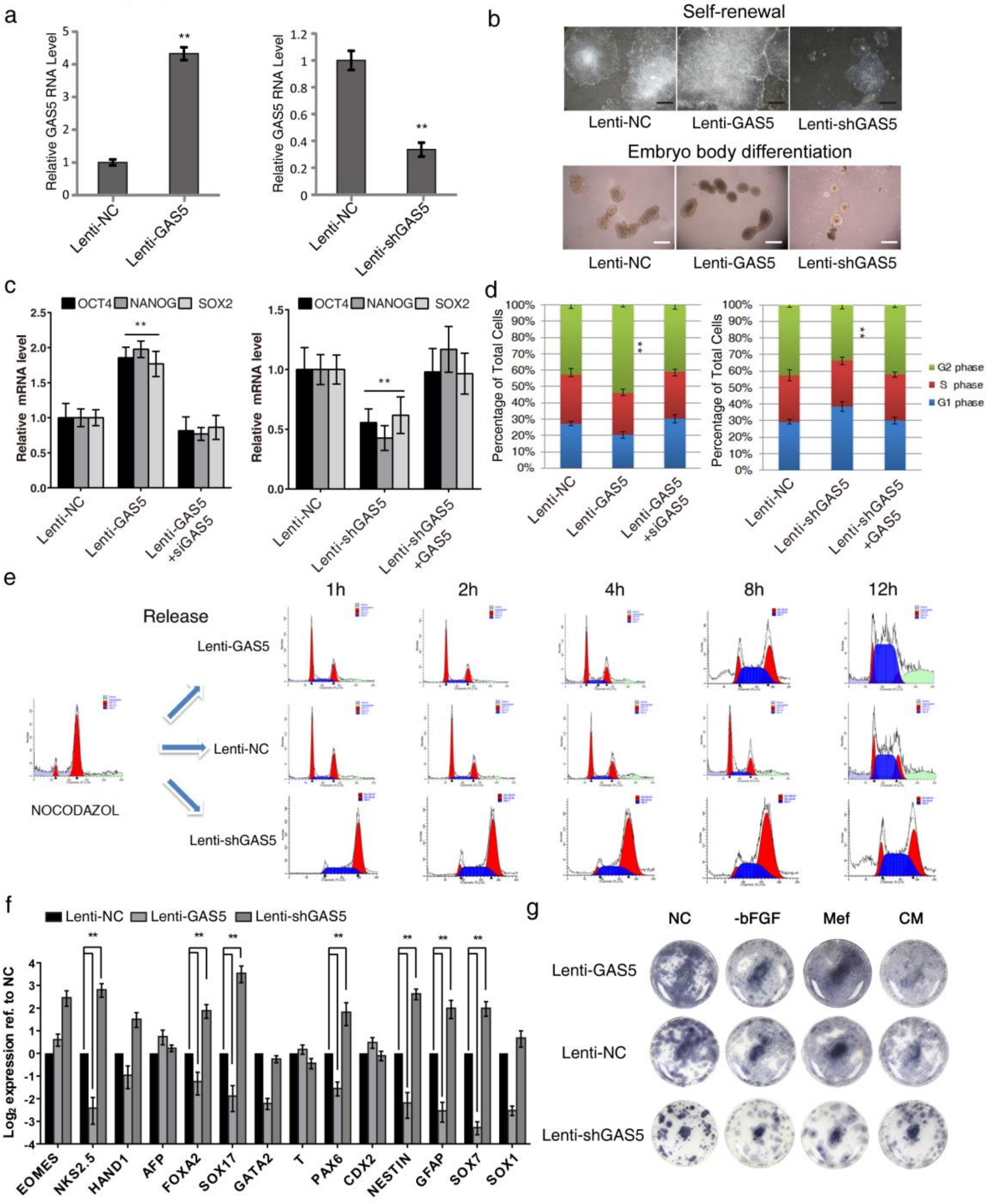

Supplementary Figure 2, related to Figure 2. GAS5 is Essential for hESC Self-renewal.

(**a**) The expressing efficacy of different stable lentivirus treated cells. **P<0.01, t-test, n=3. (**b**) The light microscopy showing the growing state of different stably lentivirus treated hESCs (upper panel). The lower panel shows the embryo bodies formed by different stably expressed hESCs. Lenti-NC represents control lentivirus, Lenti-GAS5 represents GAS5 overexpressing lentivirus, and Lenti-shGAS5 represents short

hairpin knockdown lentivirus, n=2. (**c**) QPCR analysis to measure the effect of GAS5 knockdown in GAS5 stably overexpressed hESCs, and GAS5 overexpression in GAS5 stably knockdown hESCs on the expression level of pluripotency genes. $^{**}P<0.01$, t-test, n=3. (**d**) Quantification of cell cycle analysis to show the effect of GAS5 knockdown in GAS5 stably overexpressed hESCs, and GAS5 overexpression in GAS5 stably knockdown hESCs on the proliferation of hESCs. The percentage of G2 phase cells were statistically analyzed, $^{**}P<0.01$, t-test, n=3. (**e**) Cell cycle synchronization assay (n=2) performed in different stably expressed hESCs. The cells were treated with NOCODAZOL at 100ng/ml for 12h. 2h before experiment, the cells are washed In PBS and changed for new medium. (**f**) The analysis of markers of three germ layers in different stably expressed hESCs. $^{**}P<0.01$, t-test, n=3. (**g**) Alkaline phosphatase staining of different stably expressed hESCs in different culture medium (n=2). NC represents normal hESCs culture, -bFGF represents using medium that withdraw bFGF, MEF represents using murine embryonic fibroblast (MEF) culture medium, and CM represents using conditioned hESCs medium produced by MEF. Error bars represent standard deviation of the indicated experiment replicates. RNA level of β-actin served as internal reference for qPCR.

Supplementary Figure 3. Houqi

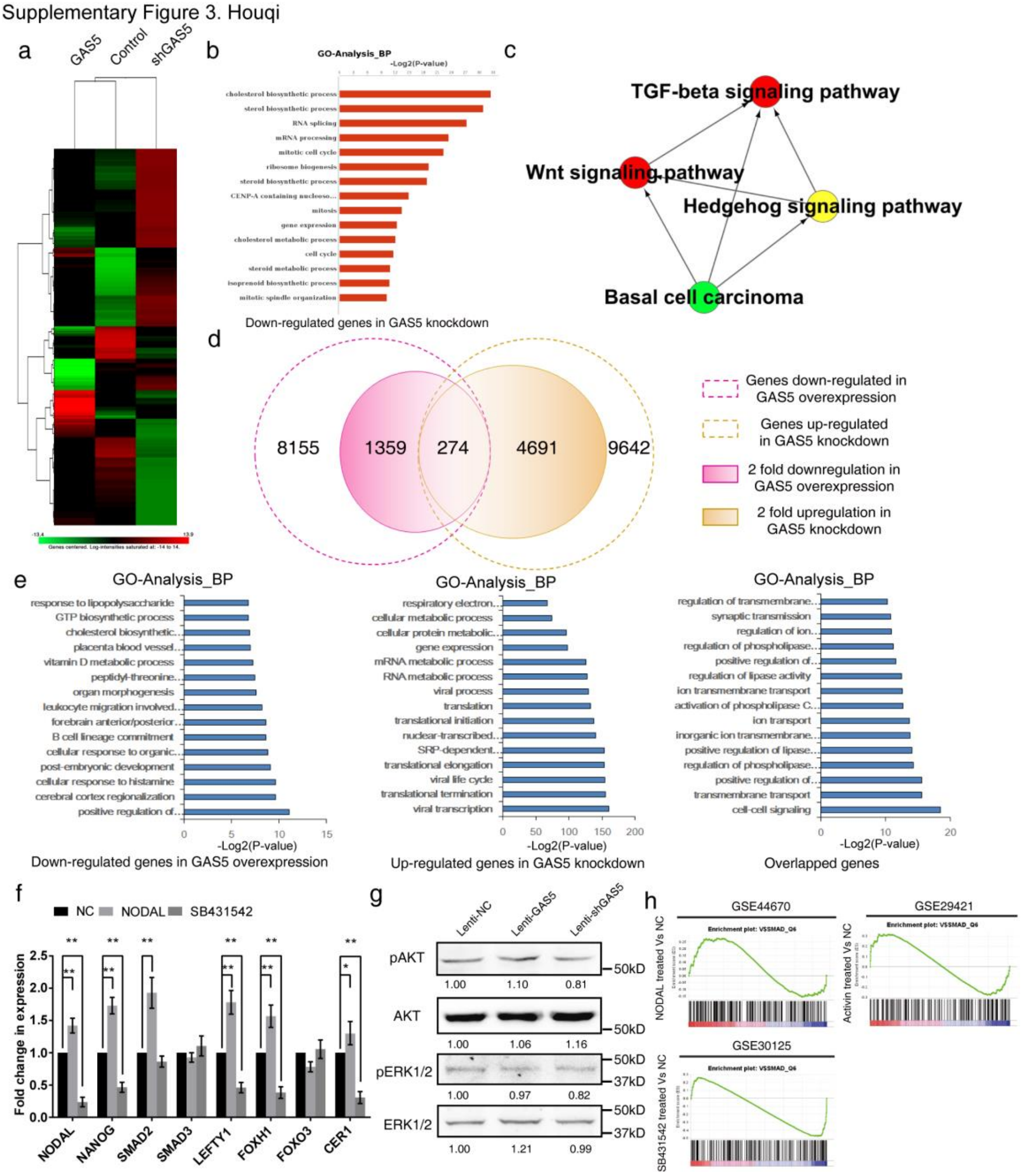

Supplementary Figure 3, related to Figure 3. Global mRNA analysis of GAS5-regulated hESCs revealed NODAL-SMAD signaling is activated by GAS5 in hESCs.

(**a**) Hierarchical clustering showing differentially expressed genes in stably GAS5 overexpressed or knockdown hESC cells. Red color indicates higher expression, whereas green color indicates lower expression. (**b**) The chart shows the Gene Ontology analysis of down-regulated genes in GAS5 knockdown group indicated in Fig. 3a. (**c**) The signaling network analysis based on the sequencing data showed that TGFβ and Wnt signaling is up-regulated during GAS5 overexpression, whereas genes

involved in basal cell carcinoma is lowered. However, genes involved in Hedgehog signaling showed bilateral change. (**d**) The graph depicts the number of inversely expressed genes with GAS5 which generated form the sequencing. (**e**) Gene Ontology analysis of the genes in inverse correlation with GAS5. Each chart represent gene groups indicated in (**d**). (**f**) The effect of rhNODAL treatment on NODAL signaling related genes. SB431542, an ALK4/7 inhibitor that blocks NODAL and Activin mediated signal transduction. ** $P<0.01$, t-test, n=3. (**g**) Western blot (n=3) showing the effect of GAS5 on the protein amount of signaling regulators AKT and ERK, and their phosphorylated form pAKT and pERK. (**h**) GSEA gene signature analysis of SMAD binding genes in microarray or high through-put sequencing data that involve NODAL or Activin treatment. SB431542, an ALK4/7 inhibitor that blocks NODAL and Activin mediated signal transduction. Related to Fig. 3g. Error bars represent standard deviation of the indicated experiment replicates. RNA level of β-actin served as internal reference for qPCR.

Supplementary Figure 4. Houqi

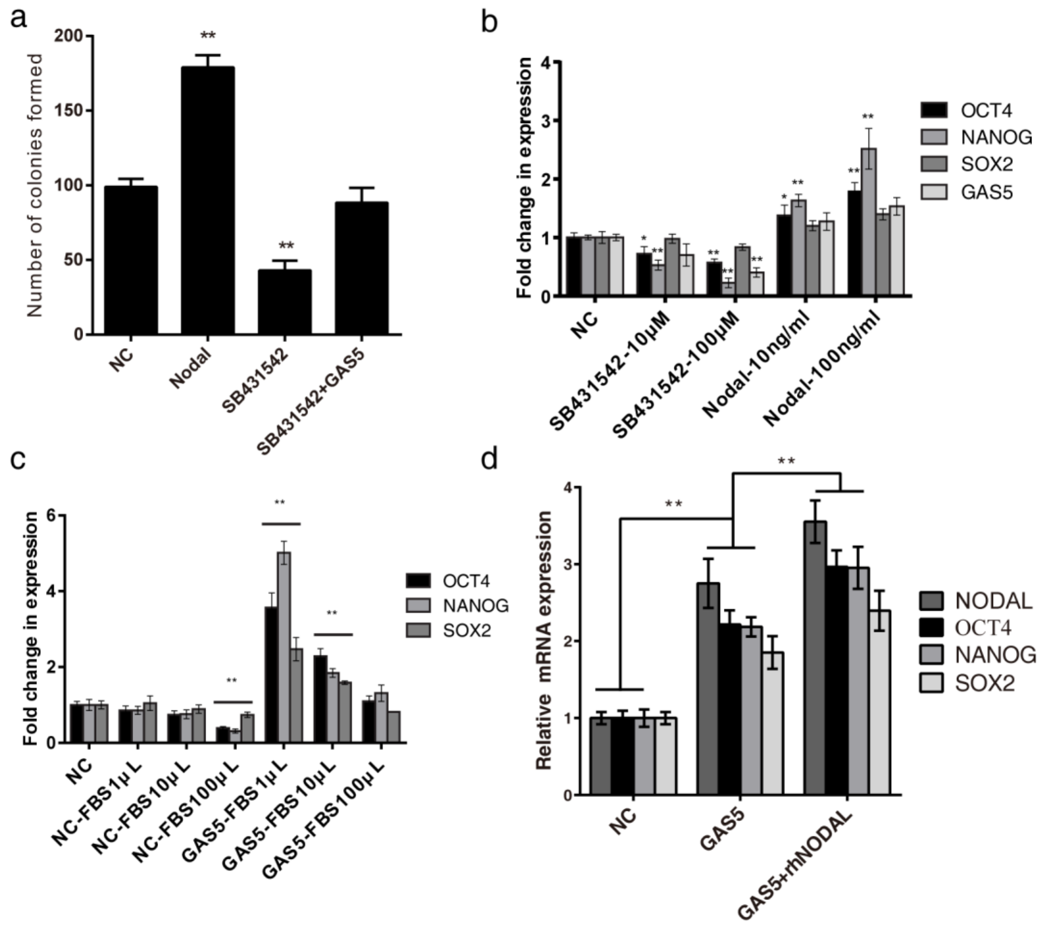

Supplementary Figure 4, related to Figure 4. NODAL-Signaling is required for the GAS5-promoted Self-renewal of hESCs.

(**a**) Quantification of colony formation assay in Fig. 4b. ***P*<0.01, t-test, n=3. (**b**) The effect of different doses of SB431542 and NODAL on pluripotency genes and GAS5 expressions in hESCs. (**c**) The analysis of the mRNA level of pluripotency genes in GAS5 overexpressed or control hESCs during different doses of fetal bovine serum (FBS) induced differentiation. The bar graph showed that increased doses of FBS (per 12-well) induced down-regulation of the expression of pluriportency genes in either GAS5 overexpressed or control hESCs. **P*<0.05, ***P*<0.01, t-test, n=3. (**d**) qPCR analysis of pluripotency genes in GAS5 or GAS5+rhNODAL treated group compared with negative control (empty vector) group. ***P*<0.01, t-test, n=3. Error bars represent standard deviation of the indicated experiment replicates. RNA level of β-actin served as internal reference for qPCR.

Supplementary Figure 5. Houqi

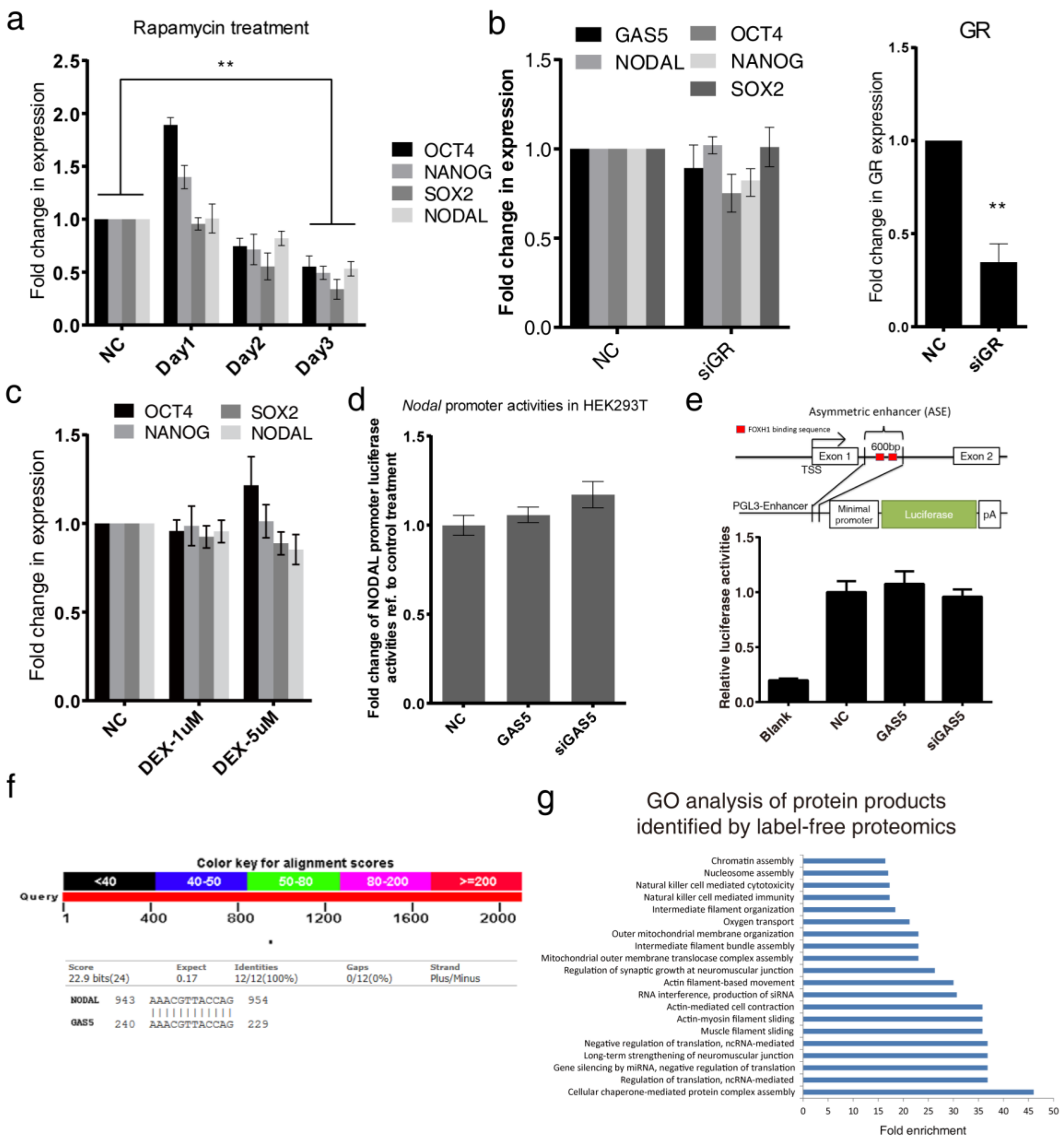

Supplementary Figure 5, related to Figure 5. NODAL is Post-transcriptionally Regulated by GAS5.

(**a**) Several reports have demonstrated the involvement of GAS5 in mTOR pathway. So we first analyzed whether mTOR pathway would have effects on GAS5 related pluripotency control using Rapamycin, an mTOR inhibitor. The qPCR analysis of the expression of pluripotency genes in Rapamycin treated hESCs at different time points. *P<0.05, **P<0.01, t-test, n=3. (**b**) Other report showed that GAS5 can directly bind Glucocorticoid Receptor (GR) to inhibit its activity. So we use GR siRNA to analyze the effect of GR on pluripotency in hESCs. The efficacy of GR siRNA-mediated knockdown (right panel), and the effect of siGR on the mRNA level of pluripotency

genes and GAS5 transcript (left panel). ***P*<0.01, t-test, n=3. (**c**) The expression change of pluripotency genes and NODAL under different doses of Dexamethasone treatment. DEX represents Dexamethasone. T-test, n=3. (**d**) Luciferase activities of NODAL promoter in HEK-293T cells with different treatment indicated. **P*<0.05, t-test, n=3. (**e**) Since report showed that enhancer in the first intron of *Nodal* gene may be more important to the expression of *Nodal* in mouse EpiSCs (resembles hESCs), we analyzed the coordinate ASE in human *Nodal* gene as indicated in the upper panel. The luciferase reporter assay showed that GAS5 modulation did not significantly influence the reporter activity, which indicates that GAS5 did not modulate NODAL expression through increasing its transcription, t-test, n=4. (**f**) Sequence alignment of GAS5 and NODAL transcript. Blast results from NCBI showed that only one region is complimentary between two transcripts and the length of which is 12bp long. Indicating there is low possibility for GAS5 and NODAL transcript to form stable RNA-RNA complimentary duplex to increase RNA stability. (**g**) GO analysis of proteins identified in MS2-GAS5 pull down products using label-free proteomics. Error bars represent standard deviation of the indicated experiment replicates. RNA level of β-actin served as internal reference for qPCR.

Supplementary Figure 6. Houqi

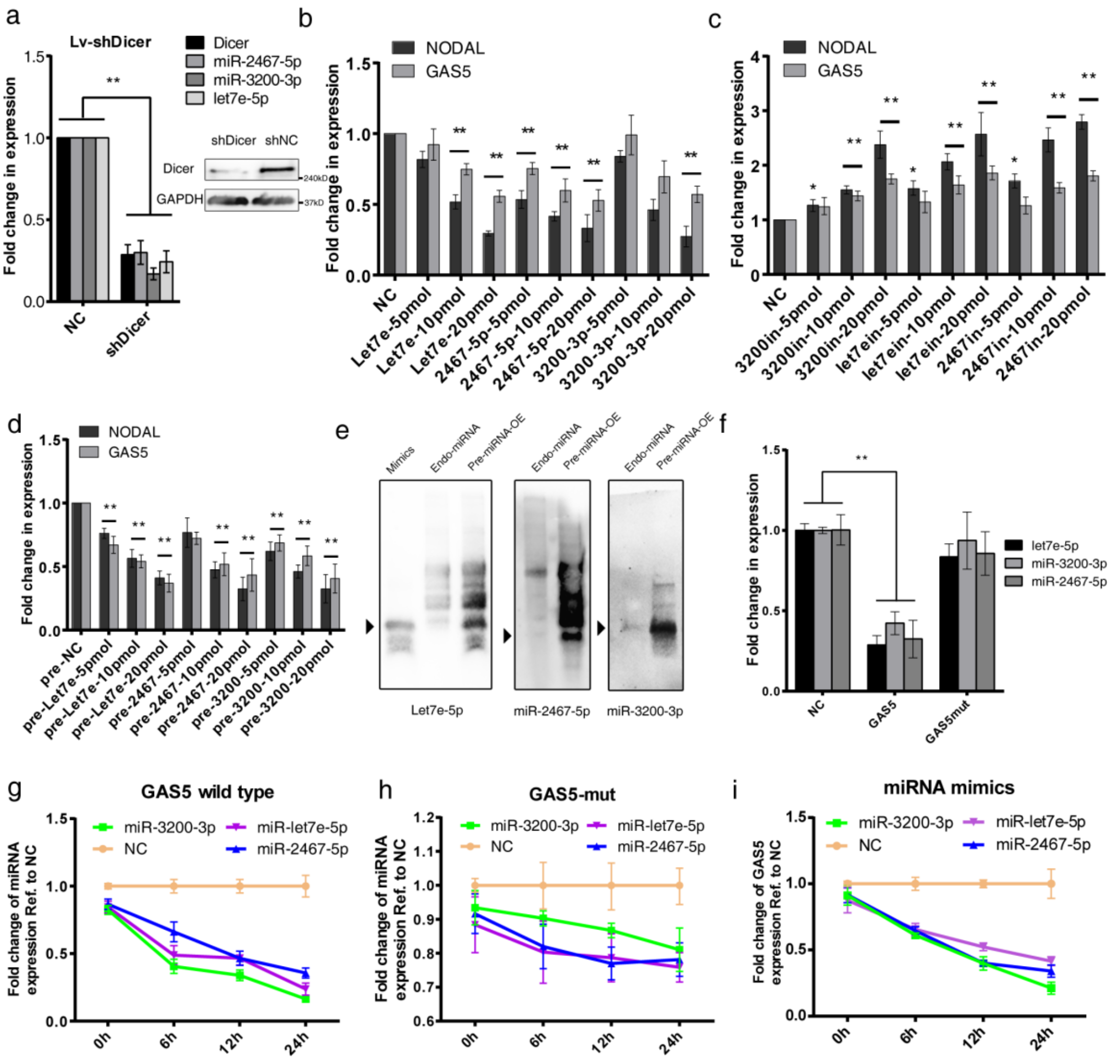

Supplementary Figure 6, related to Figure 6. Mirnome analysis revealed that GAS5 competes miR-2467, miR-3200, and Let7e with NODAL mRNA to sustain NODAL expression in hESCs

(**a**) The efficacy of Dicer knockdown by lentiviral shRNA. The results showed that Dicer along other miRNAs we tested reduced significantly upon Dicer knockdown. ***P*<0.01, t-test, n=3. (**b-c**) The effect of different doses of microRNA mimics (**b**) or inhibitors (**c**) to the mRNA level of NODAL and GAS5. The concentration here shows the amount of microRNA inhibitors or mimics used for each 12-well transfection. 3200In stands for microRNA-3200 inhibitors, and 3200, 2467 and Let7e stands for relative microRNA mimics. An Inhibitor that binds to none of these microRNAs and a microRNA mimic that differs greatly from these microRNA were used as respective negative control (NC). **P*<0.05, ***P*<0.01, t-test, n=3.. (**d**) The

effect of overexpressing different doses of precursor microRNAs to the mRNA level of NODAL and GAS5 in hESCs were detected using qPCR. Here, a scramble hairpin precursor RNA serves as negative control named as pre-NC. All other pre-mature microRNA were synthesized oligos according to miRBase (v21). ***P*<0.01, t-test, n=3. (**e**) Northern Blot analysis (n=2) of miR-3200-3p, miR-2467-5p and let7e-5p in hESCs using anti-sense probes. The Pre-miRNA lane represents total miRNAs that extracted from pre-miRNA overexpressed hESCs. Each lane is loaded with 1ug of total miRNA. (**f**) The effect of overexpressing GAS5 or GAS5-mut on the expression of miRNAs. GAS5-mut represents the miRNA binding sites mutated GAS5 transcript (Fig. 6e). (**g-h**) The degradation assay (n=3) analyzing the turnover of the indicated miRNAs upon GAS5 wild type transcript (**g**) or GAS5-mut (**h**) transcript transfection. Detection of U6 small RNAs at different time point were served as negative control (NC). (**i**) The degradation assay analyzing the turnover of the indicated miRNAs upon GAS5 transfection (n=3). A microRNA mimic that differs greatly from these microRNAs was used as negative control (NC). Error bars represent standard deviation of the indicated experiment replicates. RNA level of β-actin served as internal reference for qPCR.

Supplementary Figure 7. Houqi

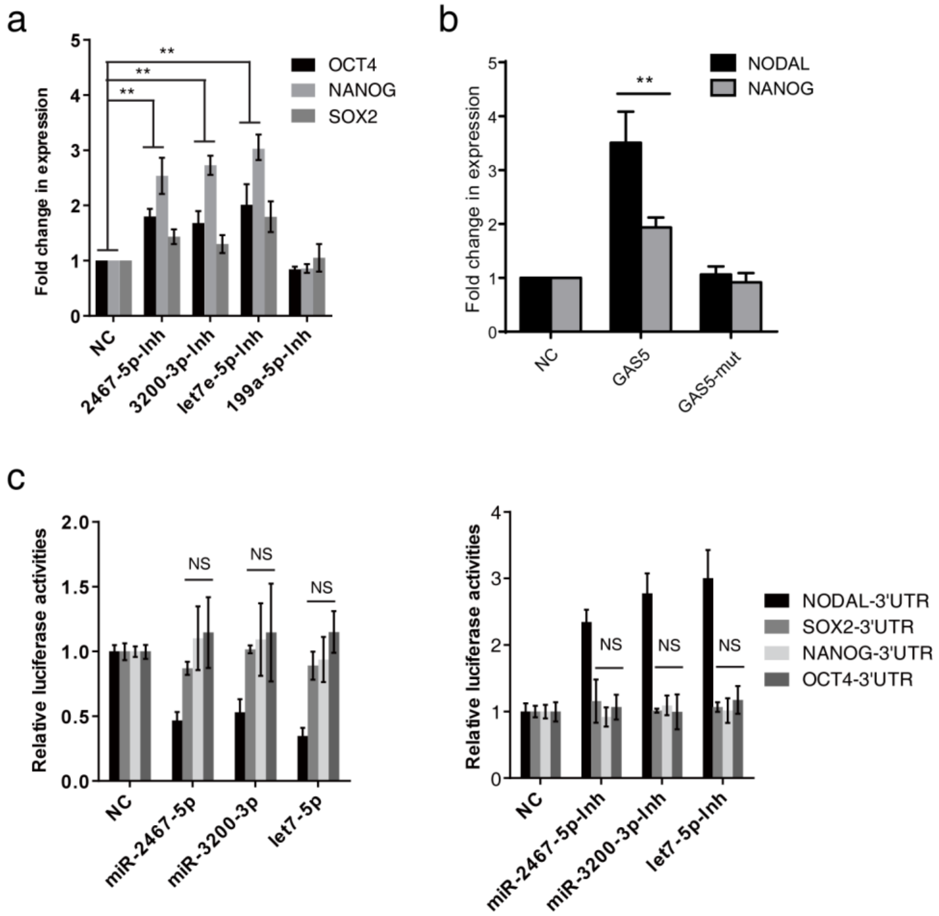

Supplementary Figure 7, related to Figure 7. MiR-2467, miR-3200, and Let7e inhibit self-renewal and attenuate GAS5-mediated NODAL signaling in hESCs

(**a**) The expression change of pluripotency genes under relative miRNA inhibition were measured using qPCR. Suffix '-Inh' represents relative microRNA inhibitors, NC represents scramble inhibitor controls. **P*<0.05, ***P*<0.01, t-test, n=3. (**b**) Relative NODAL and NANOG levels in different treatment group. ***P*<0.01, t-test, n=3. (**c**) The luciferase activities of pluripotency gene 3'UTR reporters under relative miRNA transfection (left panle) or inhibition (right panel). Suffix '-Inh' represents relative microRNA inhibitors, groups with no suffix '-Inh' represent microRNA mimic overexpression. NS, non-significant, t-test, n=4. Error bars represent standard deviation of the indicated experiment replicates. RNA level of β-actin served as internal reference for qPCR.

Supplementary Figure 8. Houqi

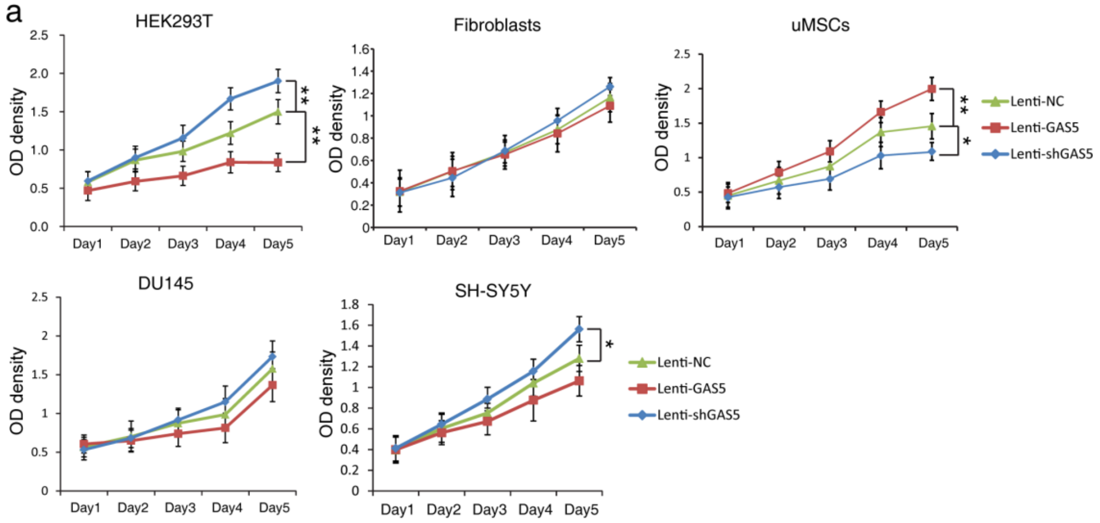

Supplementary Figure 8, related to Figure 8. GAS5 inhibited cell growth in other cell types than hESCs

(**a**) The effect of GAS5 overpression or knockdown on cell growth assessed by CCK-8 analysis in HEK-293T, Fibroblasts, uMSC, DU145 and SH-SY5Y cell lines. s.d. of three independent experiments with three replicate each time are indicated. **P*<0.05, ***P*<0.01, ANOVA was used to measure the significance of each groups.

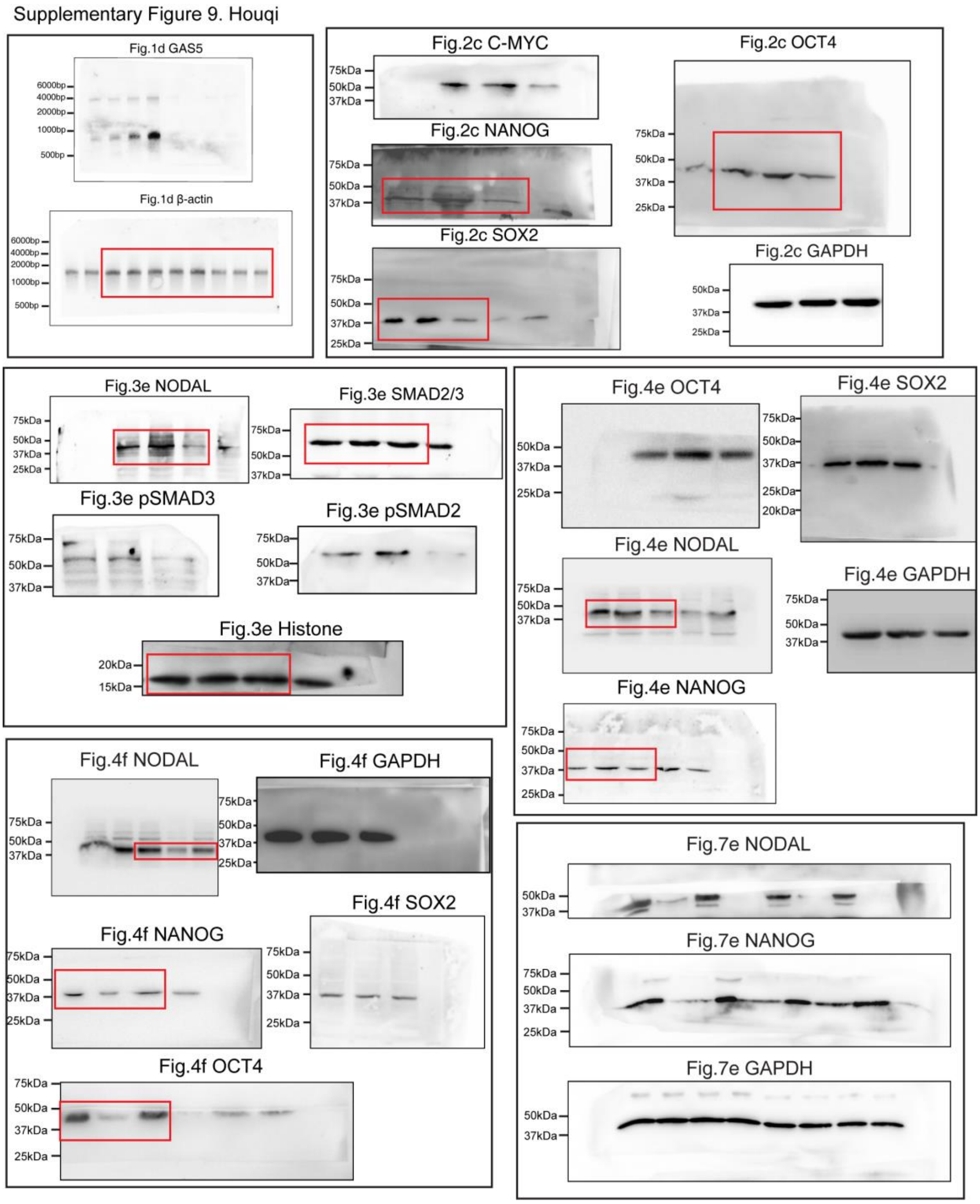

Supplementary Figure 9.

The uncropped images of blots shown in the main figures (Red boxes indicated the area shown in the main figures).
